# Ginkgo Biloba Bioactive Phytochemicals against Age-Related Diseases: Evidence from a Stepwise, High-Throughput Research Platform

**DOI:** 10.3390/antiox13091104

**Published:** 2024-09-12

**Authors:** Yuming Yuan, Xiaoyan Xiang, Xuejun Jiang, Yingju Liu, Ming Zhang, Luyang Lu, Xinping Zhang, Xinyi Liu, Qunyou Tan, Jingqing Zhang

**Affiliations:** 1 College of Pharmacy, Chongqing Medical University, Chongqing 400016, China; yuanyuming@stu.cqmu.edu.cn (Y.Y.); xiangxiaoyan0709@163.com (X.X.); 20222110046@stu.gzucm.edu.cn (X.J.); liuyingju@cqmu.edu.cn (Y.L.); zhangxinping@stu.cqmu.edu.cn (X.Z.); 2023121833@stu.cqmu.edu.cn (X.L.); 2 Department of Thoracic Surgery, University-Town Hospital of Chongqing Medical University, Chongqing 401331, China; zhangming@hospital.cqmu.edu.cn; 3 College of Pharmacy, Southwest Minzu University, Chengdu 610041, China; luluyang@swun.edu.cn

**Keywords:** whole ginkgo biloba seed powder, anti-aging, anti-atherosclerosis, anti-fatigue, network pharmacology

## Abstract

The seeds of ginkgo biloba L (GB) have been widely used worldwide. This study investigated the bioefficacies of whole GB seed powder (WGP) retaining the full nutrients of ginkgo against aging, atherosclerosis, and fatigue. The experimental results indicated that WGP lowered brain monoamine oxidase and serum malondialdehyde levels, enhanced thymus/spleen indexes, and improved learning ability, and delayed aging in senescent mice. WGP regulated lipid levels and prevented atherosclerosis by reducing triglycerides, lowering low-density lipoprotein cholesterol, increasing high-density lipoprotein cholesterol, and decreasing the atherosclerosis index. WGP improved exercise performance by reducing blood lactate accumulation and extending exhaustive swimming and climbing times, improved energy storage by increasing muscle/liver glycogen levels, and relieved physical fatigue. Network pharmacology analysis revealed 270 potential targets of WGP that play roles in cellular pathways related to inflammation inhibition, metabolism regulation, and anti-cellular senescence, etc. Protein-protein interaction analysis identified 10 hub genes, including FOS, ESR1, MAPK8, and SP1 targets. Molecular docking and molecular dynamics simulations showed that the bioactive compounds of WGP bound well to the targets. This study suggests that WGP exerts prominent health-promoting effects through multiple components, targets, and pathways.

## 1. Introduction

Aging is a process characterized by degenerative damage to organs and their functions [1,2]. Aging is the single greatest risk factor for many age-related diseases, such as atherosclerosis, neurodegeneration, and dementia, and it is an important risk factor for many age-related diseases, such as chronic fatigue syndrome, hypertension, and osteoporosis [3,4]. Atherosclerosis is a chronic inflammatory disease characterized by the formation of arterial wall plaques containing inflammatory cells and lipids [5]. Advanced age is an independent risk factor for atherosclerosis [6,7]. Atherosclerosis is also a major cause of cardiovascular diseases (CVDs), such as myocardial infarction, cerebral infarction, and stroke [8]. Age-related cardiovascular disease risk factors and cognitive disorders influence each other. Fatigue is a symptom or comorbidity of aging or age-related disorders [9]. Fatigue is responsible for the deterioration of the quality of life and prognosis of diseases [10,11]. Considering safety and effectiveness, the potential efficacy of natural products in the prevention of aging, atherosclerosis, and fatigue has attracted wide attention.

Ginkgo biloba L (GB) has existed for 200 million years, and it is regarded as a “living fossil” [12]. GB possesses many bioactive constituents, including flavonoids, alkylphenols, and polysaccharides, and it exerts various pharmacological effects [13,14]. The leaf extract of GB has been widely used to solve different diseases, such as Alzheimer’s disease, CVDs, and cerebral insufficiency [15,16,17]. The leaf extract also has gained increasing attention as an anti-aging therapy to slow aging and aging-associated disorders [18,19]. In comparison with a host of studies about the leaf extract, there have been few studies about ginkgo seed or seed exocarp [20,21], which possesses the same bioactive constituents as that found in the leaf (such as flavonoids, terpene trilactones, and alkylphenols) and may exhibit similar treatment efficacy. However, the toxic ingredients in the raw seed of GB, such as ginkgotoxin and ginkgolic acid, have hindered its application [22].

Network pharmacology provides a useful approach to systematically unveiling the underlying mechanisms of a natural product containing multiple components through overcoming the “one disease-one target-one drug” problem [23]. This approach explores the overall relationship between an active multi-ingredient and a disease by discovering multiple potential targets and pathways [24]. Molecular docking and dynamics simulations predict the binding mode, the affinity of receptor–ligand complexes, and the structural stability [25].

**Figure 1:**
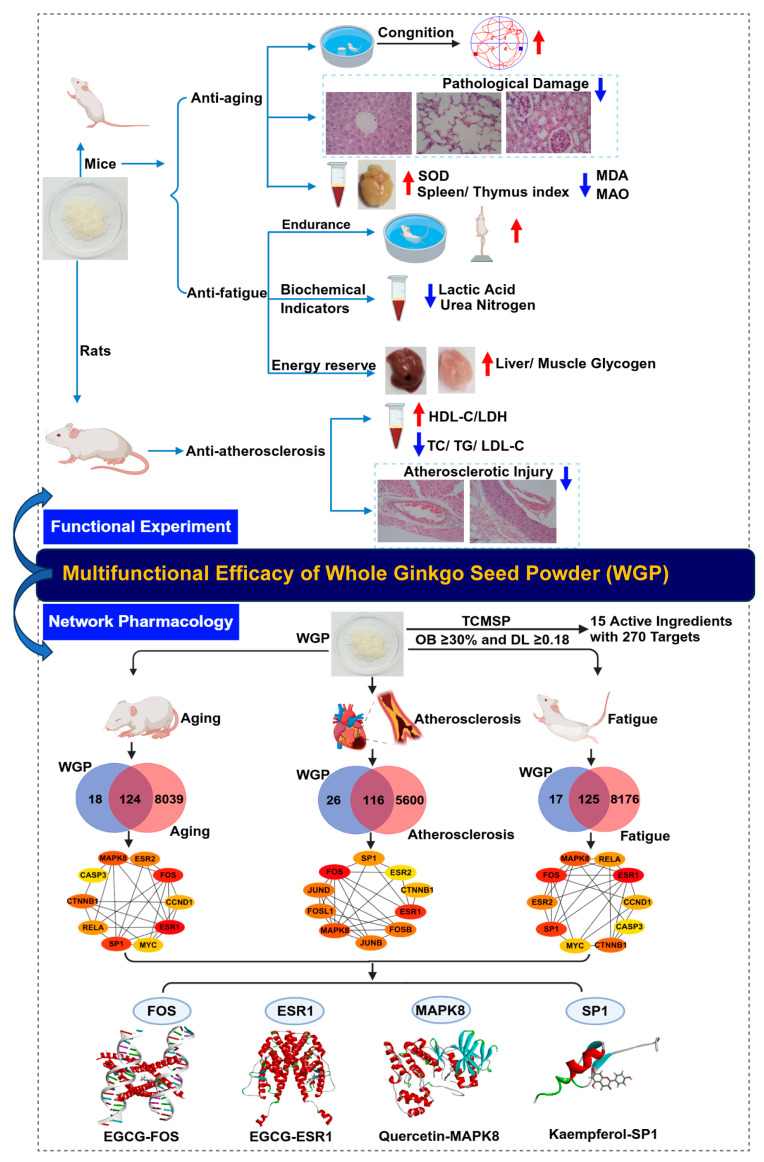
Schematic diagram depicting the experimental studies and network pharmacological analysis to assess the multifunction efficacy of whole ginkgo biloba L powder.

This study investigated the effects of whole GB seed powder (WGP) prepared using an authorized national patented technology without the addition of any chemical additives on delaying aging, preventing atherosclerosis, and relieving physiological fatigue. We measured the biochemical parameters of aging or fatigued mice treated with WGP, and we determined the lipid levels and atherosclerosis index (AI) of high-fat-fed rats treated with WGP. Furthermore, we applied network pharmacology to analyze the target genes of active ingredients in WGP. Protein–protein interaction (PPI) analysis identified the top 10 target genes of WGP, while gene ontology (GO) and the *Kyoto Encyclopedia of Genes and Genomes* (KEGG) cellular pathway systems provided the biological pathways of WGP and diseases. The molecular docking technique further indicated the binding mode of important active components of WGP with major core targets at the molecular level. This study aimed to evaluate the multi-functions of WGP and understand their underlying mechanisms (Figure 1).

## 2. Materials and Methods

### 2.1. Materials

D-galactose (D-gal) (purity ≥ 98%) was provided by Dingguo Changsheng Biotechnology Co., Ltd. (Beijing, China). Vitamin E (VE) soft capsules (batch No. 10874004) were obtained from Sinopharm Xingsha Pharmaceutical Co., Ltd. (Xiamen, China). Vitamin D3 (300,000 U/mL) was obtained from Harbin Zhuohongda Animal Medicine Factory (Harbin, China). Simvastatin (Sim) tablet (1 mg/tablet) was obtained from Harbin Pharmaceutical Group Sanjing Mingshui Pharmaceutical Co., Ltd. (Suihua, China). A high-fat diet (HFD, 3% cholesterol, 0.2% pig bile salt, 15% granulated sugar, 20% lard, and 61.8% high protein pellet diet) was bought from Shengmin Experimental Animal Farm (Nanjing, China). Detection kits for blood urea nitrogen (BUN), blood lactic acid (BLA), superoxide dismutase (SOD), malondialdehyde (MDA), monoamine oxidase (MAO), muscle glycogen (MG), and liver glycogen (LG) were purchased from Nanjing Jiancheng Biology Engineering Institute (Nanjing, China). Detection kits for total cholesterol (TC), triglyceride (TG), high-density lipoprotein cholesterol (HDL-C), and low-density lipoprotein cholesterol (LDL-C) were purchased from Mindray Medical International Ltd. (Shenzhen, China). The other reagents used in this study were of analytical grade.

### 2.2. Preparation of WGP

Raw GB material was collected in Bali Village, Jianggang Town, Dongtai, China (32.525229° N 120.462100° E). The whole plant was identified as Ginkgo biloba L by Professor Fuliang Cao from the Co-Innovation Center for Sustainable Forestry in Southern China, Nanjing Forestry University. WGP was obtained from Jiangsu Dongtai Jieer Ginkgo Technology Co., Ltd. (Dongtai, China). WGP was prepared using an authorized national patented technology (CN104432272A, China). In brief terms, fresh GB seeds were removed from their hard outer shell and inner endothelium. And then, they were pulped and filtrated with a 100-mesh sieve. The obtained slurry was gelatinized at 150 °C for 60 s and then subjected to dehydration and drying at 150 °C and under 0.5 mPa vapor pressure. The dried GB seed aggregate was crushed and screened with a 5-mesh sieve to obtain the WGP.

### 2.3. Animals

Young (28 d old) and adult Kunming mice (300 d old) were obtained from the Laboratory Animal Center of Chongqing Medical University (Chongqing, China). The animals were kept at 20 ± 2 °C with a relative humidity of 55 ± 5% and a 12 h light/12 h dark cycle. All in vitro and in vivo studies were conducted in accordance with the Guidance for Animal Experiments established by Chongqing Medical University. The protocol was approved by the Committee on the Ethics of Animal Experiments of Chongqing Medical University (SCXK (Yu) 2018-0003). All authors read and approved the manuscript.

### 2.4. Anti-Aging Activity Assay

#### 2.4.1. D-Gal-Induced Aging Mice and Treatments

The young (*n* = 10, half male and half female, group A) and adult (*n* = 60, half male and half female) Kunming mice were allowed free access to feed and water during the whole experimental period. 

After adapting to the environment for 7 d, the mice in group A (negative control, young and normal) and in group B (negative control, adult and normal, *n* = 10) were treated with a standard diet and water during the experimental period. The mice in groups C-G (*n* = 10 per group) were subcutaneously injected with D-gal (1 g/kg mice/d) to induce aging. The mice in group C constituted the negative control (old, the model group). The mice in groups D-G were orally administrated with a therapeutic drug (VE, 0.1 g/kg mice/d) and WGP (2, 4, and 6 g/kg mice/d, corresponding to WGP-L, -M, and -H), respectively. The mice were fed for 28 d and subjected to the Morris water maze test.

#### 2.4.2. Morris Water Maze Test

A water maze (120 cm in diameter and 50 cm in height, ZS-001, Beijing Kedi Zhongchuang Co., Ltd., Beijing, China) filled with water (20 ± 1 °C) was divided into four quadrants. A mobile platform (6.5 cm in diameter and 15 cm in height) submerged 1 cm under the water surface was placed in the first quadrant [26]. Each mouse was trained to find the hidden platform from the entry point of each quadrant in the first five days. If the mouse found the platform within 90 s, it would be allowed to stay there for 15 s, and the escape latency time was recorded as 15 s. If the mouse could not find the platform within 90 s, it would be guided and remained there for 30 s, and the escape latency time was recorded as 90 s. A spatial probe trial was performed by removing the hidden platform on the seventh day. The mouse was released from the same start point in the third quadrant and allowed to freely swim for 120 s. The swimming-motion trail, platform-crossing frequency, swimming distance, and time in the first quadrant for each mouse were recorded and analyzed.

#### 2.4.3. Biochemical Parameters in Serum and Tissue

After a behavioral investigation, blood samples and spleen, thymus, heart, liver, lung, kidney, and brain tissues were collected. The blood samples were centrifuged at 1000× *g* for 10 min at 4 °C to isolate serum. All the samples were stored at −80 °C for further analyses. The brain was weighed and homogenized with cool saline in an ice bath after cleaning. The tissue homogenate was then centrifuged at 1000× *g* at 4 °C for 10 min. The supernatant was collected for biochemical analyses. The serum SOD, MDA, and brain MAO activity were measured with corresponding detection kits. The spleen and thymus indexes were calculated using Equations (1) and (2).

Spleen index (%) = *W*_spleen_/*W*_body_ × 100% (1)

Thymus index (%) = *W*_thymus_/*W*_body_ × 100% (2)

where *W*_spleen_, *W*_thymus_, and *W*_body_ refer to the spleen, thymus, and body weight, respectively.

#### 2.4.4. Histopathological Analysis

The livers, lungs, and kidneys of the mice were fixed with 4% paraformaldehyde for 24 h and embedded in paraffin. The cut sections (4 μm-thick) using a microtome (Leica, Wetzlar, Germany) were stained with hematoxylin and eosin (H&E). The histopathological changes in liver and lung tissues were observed via optical microscopy (Nikon, Tokyo, Japan).

### 2.5. Anti-Atherosclerosis Activity Assay

#### 2.5.1. Diet and Treatment

Healthy Sprague Dawley male rats weighting 200 ± 20 g (6~8 months) were obtained from the Chongqing Laboratory Animal Center of Chongqing Medical University (Chongqing, China). The rats were housed under standard conditions. After being fed a normal diet for 7 d, the rats in groups B-F (*n* = 10 per group) were fed a high-fat diet via oral administration (free access to feed) and vitamin D3 via intraperitoneal injection (vitamin D3 is also called cholecalciferol; 600,000 IU/kg rat was intraperitoneally injected for the first 3 d) for 70 d to establish an early atherosclerosis model. The rats in group A (negative control) were fed a normal diet. The rats in group B (positive control) received no treatment. The rats in group C were given Sim at an oral dose of 1 g/kg rat. The rats in groups D-F were fed WGP at doses of 0.5, 1.0, and 1.5 g/kg rat/d. All rats were weighed every 3 d for 70 d.

#### 2.5.2. Serum Chemical Analysis

The blood samples were collected at 70 d and centrifuged at 1000× *g* for 10 min. The upper serum was stored at −80 °C for further analysis. The TC, TG, HDL-C, and LDL-C levels were determined using an Automatic Biochemical Analyzer BW-200 (Bioway, Yantai, China). The AI value was calculated using the following Formula (3).

AI (%) = (L_TC_ − L_HDL-C_)/L_HDL-C_ × 100% (3)

where L_TC_ and L_HDL-C_ referred to the TC and HDL-C levels, respectively.

#### 2.5.3. Histopathological Examination

The fresh aorta and heart were cleaned and fixed with 4% paraformaldehyde. After 24 h, the samples were embedded in paraffin and cut into 5-μm slices using a microtome (Leica, Germany) for further H&E staining. The pathological changes were observed with optical microscopy (Nikon, Japan).

### 2.6. Anti-Fatigue Activity Assay

#### 2.6.1. Animals and Experimental Design

Forty healthy male Kunming mice (28 d old, 20 ± 2 g) were procured from Chongqing Laboratory Animal Center, Chongqing Medical University (Chongqing, China). The animals were housed at standard animal room temperature (20 ± 2 °C) with 50 ± 10% humidity and a 12 h day-night cycle. The mice were allowed access to experimental chow and water freely. They were acclimatized to the experiment for 7 d prior and then randomly divided into four groups (*n* = 10). The rats in group A (negative control) were fed a normal diet. The rats in groups B-D were fed WGP at doses of 2, 4, and 6 g/kg mice/d for 28 d. All mice were raised for 28 d and used for the following antifatigue experiments.

#### 2.6.2. Climbing Test

The climbing test was performed based on the previous experiment [27]. Thirty minutes after the final oral administration, the mice were placed on a glass rod (40 cm in height and 8 mm in diameter) to cause their muscles to be in a state of tension. The climbing test was stopped when the mice fell from the glass bar because of muscle fatigue. The total climbing period was calculated as the sum of three records.

#### 2.6.3. Exhaustive Swimming Test

The exhaustive swimming test was performed as described previously [28] on the second day after the climbing test. In brief terms, 60 min after the last treatment, the mice had a load (5% of their body weight) tied onto their tail and were placed in a swimming pool filled with 40-cm-deep water (30 ± 1 °C). The whole time was recorded from the timepoint that the mice were put inside the pool to the endpoint at which the mice were exhausted (the timepoint when the mice could not rise to the surface of the water for more than 10 s).

#### 2.6.4. Measurement of Blood Biochemical Parameters and Tissue Glycogen

The mice were sacrificed after swimming freely for 90 min on the second day for an exhaustive swimming test. Their blood was collected to prepare serum via centrifugation at 1000× *g* for 10 min. The liver and muscle (the quadriceps femoris of the hind legs) were taken immediately from the mice. Serum and organs were frozen at −80 °C for subsequent tests. The BUN and BLA levels were determined using commercial kits. A certain weight of cleaned organ was mixed with 9 times the volume of saline and milled to obtain tissue homogenate. MG and LG were measured using commercial kits. 

### 2.7. Network Pharmacological Analysis

#### 2.7.1. Active Compound Screening and Potential Targets’ Identification

All the bioactive ingredients of WGP were collected from the TCMSP database (https://www.tcmsp-e.com/#/home, accessed on 11 June 2024) [29,30]. To ensure their metabolic potential in vivo, the obtained components were screened based on parameters such as oral bioavailability (OB) and drug likeness (DL). The screening criteria were OB value ≥ 30%, DL value ≥ 0.18. The potential targets of the active ingredients were then obtained from TCMSP and transformed into the corresponding gene symbols using the UniProt database (https://www.uniprot.org/, accessed on 11 June 2024).

#### 2.7.2. Identification of Target Genes 

Disease targets were collected from the GeneCards database (https://www.genecards.org/, accessed on 11 June 2024), the Online Mendelian Inheritance in Man database (OMIM, https://omim.org/, accessed on 11 June 2024), the Therapeutic Target Database (TTD, https://db.idrblab.net/ttd/, accessed on 11 June 2024), and the DisGeNET database (http://www.disgenet.org/, accessed on 11 June 2024) [31]. Subsequently, all search results were compiled, summarized, and deduplicated to create target libraries for age, AS, and fatigue.

#### 2.7.3. Protein-Protein Interaction Network Analysis

The overlapping target genes between potential active compounds and diseases were analyzed using Venn diagrams. After that, a WGP compound-target-disease network diagram was constructed using the Cytoscape 3.9.1 software. The STRING database (https://string-db.org, accessed on 11 June 2024) was used to obtain the protein-interaction relationship of the overlapping targets between ingredient targets and disease targets. The organism was set to “Homo sapiens”, and a confidence level was set to >0.9. The PPI network was established using the Cytoscape 3.9.1 software, and the top 10 core targets were screened with the CytoHubba plugin [32].

#### 2.7.4. GO and KEGG Pathway Analysis

The overlapping genes associated with both WGP active compounds and diseases were imported into the DAVID database (https://david.ncifcrf.gov, accessed on 11 June 2024), and the species “Homo sapiens” was selected for GO and KEGG enrichment analysis [33]. The results were screened according to the criterion of the False Discovery Rate (FDR) < 0.05, the *p*-values were ranked from smallest to largest, and the top 10 results were selected. Then, advanced bubble plots were drawn for a visual presentation of GO and cellular pathway enrichment results. The GO analysis results were sorted and visualized separately for the biological process (BP), cellular component (CC), and molecular function (M) categories.

#### 2.7.5. Molecular Docking Analysis

According to the network of WGP compound-target-disease, the top 3 key active ingredients were screened, and the 3D structure files of the compounds were downloaded from the PubChem database (https://pubchem.ncbi.nlm.nih.gov, accessed on 15 June 2024). Three-dimensional protein crystal structures were downloaded from the RCSB-PDB database (FOS-PDB ID: 1FOS, ESR1-PDB ID: 1A52, MAPK8-PDB ID: 3ELJ, SP1-PDB ID: 1VA1, https://www.rcsb.org, accessed on 15 June 2024). Molecular docking was performed using the Discovery Studio software (Version 2019) to validate compound-target interactions.

### 2.8. Statistical Analysis

Statistical analysis was performed with the SPSS 20.0 software, and all data were expressed as means ± standard deviations (SDs). Multiple comparisons among different groups were analyzed with a one-way analysis of variance, followed by Duncan’s test. Data were considered to indicate a significant difference at the level of *p* < 0.05.

## 3. Results

### 3.1. Anti-Aging Activity

#### 3.1.1. Assessment of Learning and Memory

**Figure 2:**
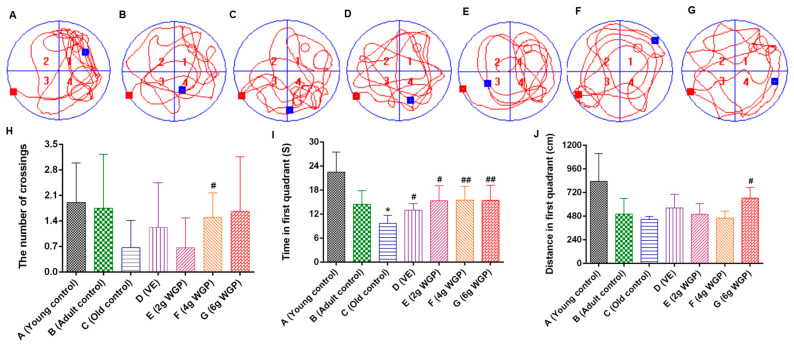
Effects of whole ginkgo powder (WGP) on (**A**–**G**) motion map and (**H**) numbers to cross the virtual platform, (**I**) time in the first quadrant, (**J**) distance in the first quadrant in the water maze test for D-galactose (D-gal)-induced aging mice. Data are expressed means ± standard deviations (SDs) (*n* = 10). * and ^#^, *p* < 0.05 compared with the adult control (Group B) and old control (Group C), respectively; ^##^, *p* < 0.01 compared with e old control (Group C). Motion map of different groups A–G: Group A, young control; Group B, adult control, non-aging; Group C, old control, non-treated; Group D, vitamin E (VE) (0.1 g/kg)-treated, aging; Group E, WGP (2 g/kg)-treated, aging; Group F, WGP (4 g/kg)-treated, aging; Group G, WGP (6 g/kg)-treated, aging.

D-Gal-induced aging mice pursued a complex and untargeted moving route, and they had difficulty finding the platform (Figure 2A–G). Compared to the model-group mice, WGP increased the ability of aging mice to find the platform and enhanced their moving time and distance in the quadrant where the platform was located (Figure 2H–J). WGP improved the learning and memory ability of the aging mice. WGP-H at a high dose seemed to have the best efficacy.

#### 3.1.2. Effects of WGP on the Spleen, Thymus, and Other Organ Indexes

**Figure 3:**
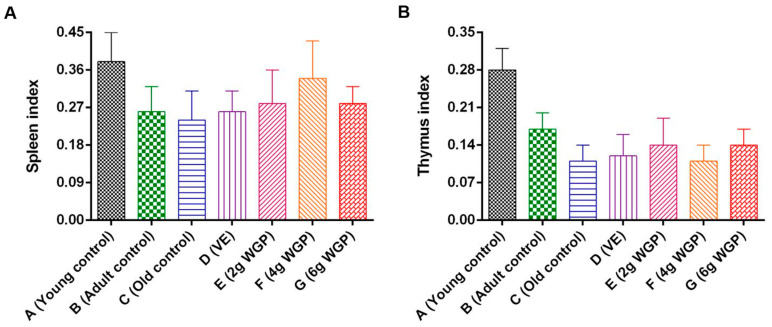
Effects of WGP on (**A**) spleen index and (**B**) thymus index in different treatment groups. Data are expressed means ± SDs (*n* = 10).

Compared to the old control, D-gal-induced aging mice had lower spleen and thymus indexes (Figure 3). However, the mice treated with different doses of WGP had increased spleen and thymus indexes. WGP-M at a middle dose obtained the best efficacy in increasing the spleen index, while the thymus index was enhanced most in the mice treated with WGP-L at a low dose and WGP-H. However, there were no differences in the spleen index and thymus indexes among the three treatment groups. In addition, there were no statistically significant differences among the heart, liver, lung, kidney, or brain indexes of the controls and/or the WGP-treated groups (Figure S1).

#### 3.1.3. Effects of WGP on Biochemical Parameters in Serum and Brain

**Figure 4:**
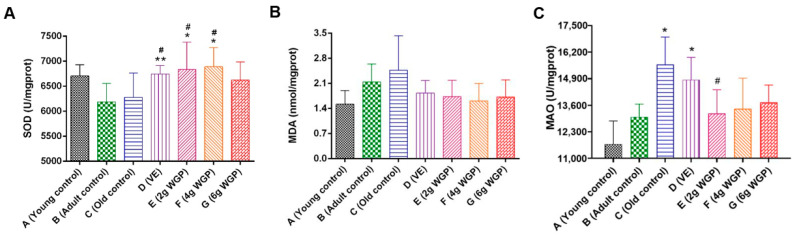
Effects of WGP of (**A**) superoxide dismutase (SOD) levels, (**B**) serum malondialdehyde (MDA), and (**C**) brain monoamine oxidase (MAO). Data are expressed means ± SDs (*n* = 10). * and ^#^, *p* < 0.05 compared with the adult control (Group B) and the old control (Group C), respectively; **, *p* < 0.01 compared with the adult control (Group B) and the old control (Group C).

Compared to the adult control, serum MDA and brain MAO were enhanced in the D-gal-induced aging mice (i.e., old control) (Figure 4). After treatment with different doses of WGP, compared to the old control, the serum SOD levels of the aging mice increased, while the serum MDA and brain MAO levels decreased. In addition, a significant reduction in brain MAO was observed in the mice treated with WGP-L. Among the three WGP-treated groups, reductions in MDA were mostly observed in the mice treated with WGP-M.

#### 3.1.4. Histopathological Changes

**Figure 5:**
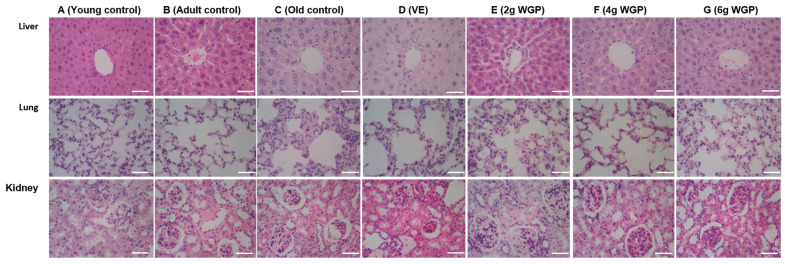
Effects of WGP on the liver, lung, and kidney of D-gal-induced aging mice. The histological changes are revealed via hematoxylin and eosin (H&E) staining (400×). Scale bar = 50 µm. Control 1, 60-d mice; Control 2, 300-d mice.

Compared to the young control, the D-gal-induced aging mice exhibited obvious changes: the liver cells had vacuoles and fat droplets and became disordered; the lung alveolar walls were markedly thickened, and their structures were destroyed; and the renal capsular space was enlarged. Encouragingly, the pathological changes were ameliorated after treatment with WGP at different doses (Figure 5).

### 3.2. Anti-Atherosclerosis Activity

#### 3.2.1. Effect of WGP on the Biochemical Parameters of HFD Rats

**Figure 6:**
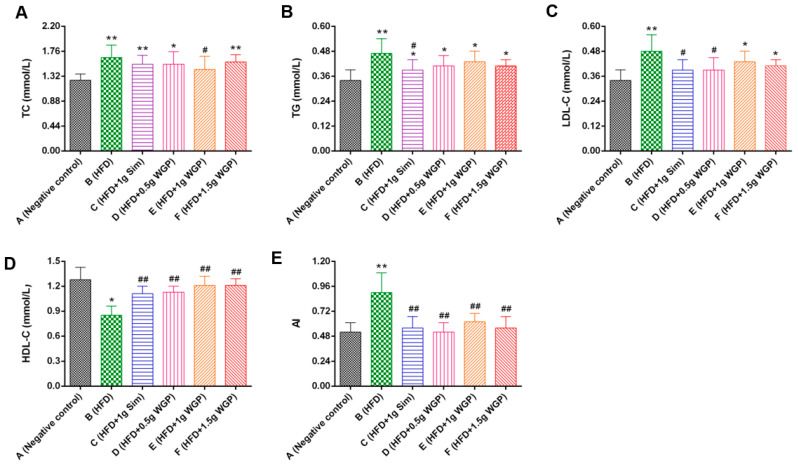
Effects of WGP on (**A**–**D**) serum biochemical lipid parameters and (**E**) atherosclerosis index (AI) in rats. Data are expressed means ± standard deviations (SDs) (*n* = 10). * and ^#^, *p* < 0.05 compared with the negative control (Group A) and high-fat diet (HFD)-fed mice (Group B), respectively; ** and ^##^, *p* < 0.01 compared with the negative control (Group A) and high-fat diet (HFD)-fed mice (Group B). Group A: negative control, old; Group B: model control, HFD-fed; Group C: treatment, treated with 1 g of simvastatin (Sim); Group D: treatment, treated with 0.5 g of WGP; Group E: treatment, treated with 1 g of WGP; Group F: treatment, treated with 1.5 g of WGP.

Compared to the negative control, the TC, TG, and LDL-C of HFD rats were significantly improved, while the HDL-C level was significantly reduced (Figure 6). When treated with WGP at different doses, the mice had reduced TC, TG, and LDL-C levels and increased HDL-C. There was a slight improvement in LDH levels (Figure S2). As shown in Figure 6E, the AI index of HFD mice was significantly increased compared to the negative control (*p* < 0.01), but the index was significantly reduced in mice treated with WGP at different doses (*p* < 0.01). However, there was no significant difference among all the treatment groups.

#### 3.2.2. Histopathological Analysis

**Figure 7:**
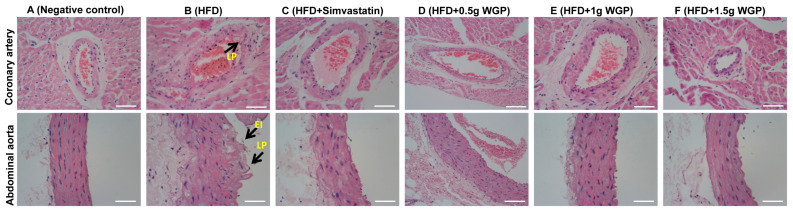
Effects of WGP on coronary artery and abdominal aorta. The histological examinations were performed via H&E staining. The lipid plaque (LP) and endothelial injury (EI) were marked in different groups (400×). Scale bar = 50 μm.

H&E staining revealed the effect of treatment on the histopathological changes in the coronary aortas and abdominal aorta (Figure 7). Compared to rats in the negative control group, aortic thickening and structural disorder, lipid plaque, and endothelial injury were found in HFD rats. Pathological changes in arteries were improved in the WGP-treated groups.

### 3.3. Anti-Fatigue Activity

#### 3.3.1. Effects of WGP on Climbing Time and Exhaustive Swimming Time

**Figure 8:**
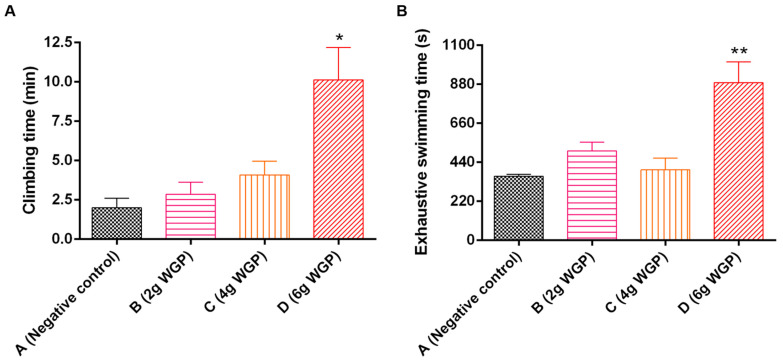
Effects of WGP on the (**A**) climbing time and (**B**) exhaustive swimming time of mice. Data are expressed as means ± standard deviations (SDs) (*n* = 10). * *p* < 0.05 and ** *p* < 0.01 compared with the negative control (Group A). Group B: treatment, treated with 2 g of WGP; Group C: treatment, treated with 4 g of WGP; Group D: treatment, treated with 6 g of WGP.

Compared to the negative control, the climbing time and exhaustive swimming time of mice were prolonged after treatment with WGP (Figure 8). The climbing time and exhaustive swimming time in mice in the WGP-H group were approximately 4 times and 1.46 times those in the negative control, respectively.

#### 3.3.2. Histopathological Changes

**Figure 9:**
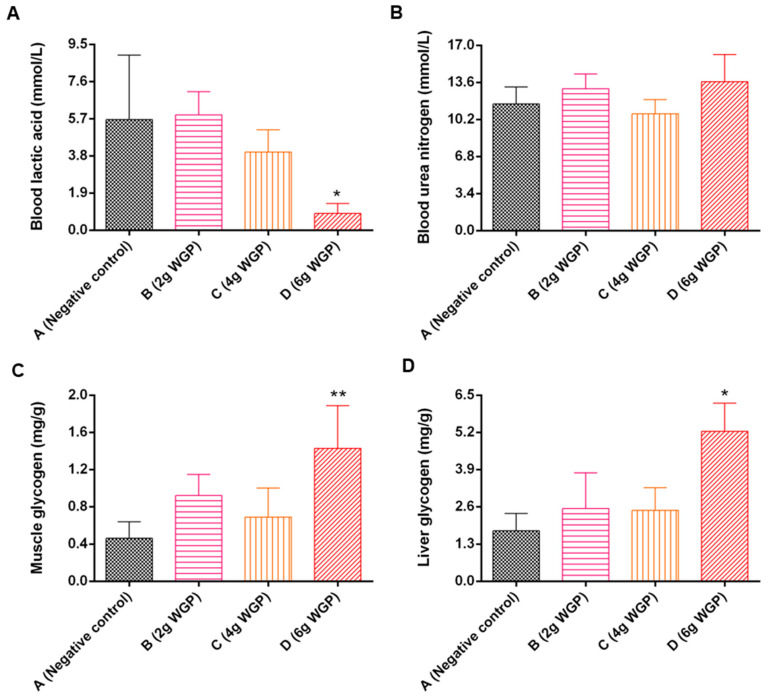
Effects of WGP on blood and tissue parameters. (**A**) Blood lactic acid (BLA); (**B**) blood urea nitrogen (BUN); (**C**) muscle glycogen (MG); (**D**) liver glycogen (LG) in mice. Data are expressed as means ± standard deviations (SD) (*n* = 10). * *p* < 0.05 and ** *p* < 0.01 compared with the negative control (Group A). Group B: treatment, treated with 2 g of WGP; Group C: treatment, treated with 4 g of WGP; Group D: treatment, treated with 6 g of WGP.

Compared to the negative control, the BLA level of mice treated with WGP-H significantly decreased (Figure 9), while the BUN level slightly changed, and the LG and MG contents significantly increased by 2.10-fold and 1.98-fold, respectively.

### 3.4. Network Pharmacology

#### 3.4.1. Screening of Active Compounds and Potential Targets

**Figure 10:**
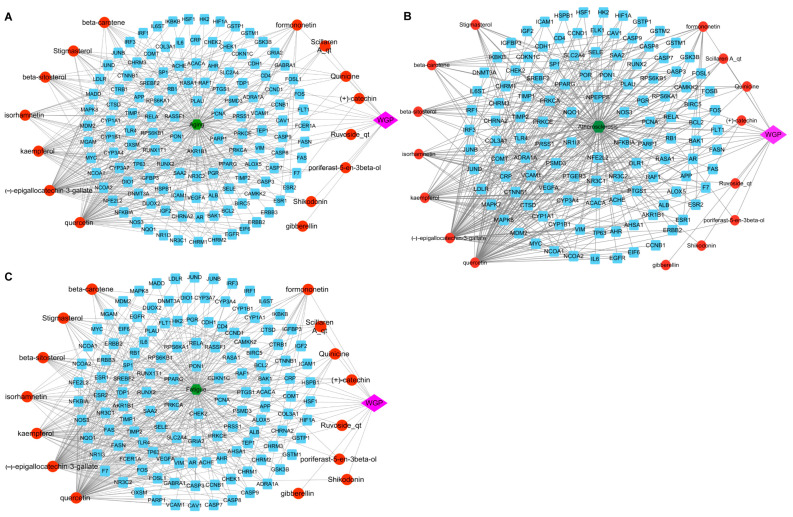
Relationships of active components and disease targets. The WGP compounds-targets-aging networks include the WGP compounds-targets-aging network (**A**), WGP compounds-targets-atherosclerosis network (**B**), and WGP compounds-targets-fatigue network (**C**). The green hexagon represents the disease, the round blue rectangle represents the target of the WGP compound, the red circle represents the WGP active ingredient compound, the edges represent the interactions between the nodes, and the dark pink diamond represents the WGP formula.

The active components of WGP were collected through the TCMSP database. There were 15 bioactive compounds that met the set parameters, which were OB ≥ 30% and DL ≥ 0.18 (Table S1). After duplicate targets were removed, 270 targets of WGP bioactive components were obtained from TCMSP and converted into the corresponding genes using the UniProt database (Table S2). We constructed a WGP compound-target-disease network (Figure 10) and analyzed the topological properties of the network using CytoNCA, a plugin in Cytoscape. Three parameters (degree, betweenness centrality, and closeness centrality) were set to distinguish the key active ingredients of the WGP. The top three key active ingredients were identified as quercetin, (-)-epigallocatechin-3-gallate (EGCG), and kaempferol, based on the scores ranked from largest to smallest.

#### 3.4.2. Target Genes of Aging, Atherosclerosis, and Fatigue

**Figure 11:**
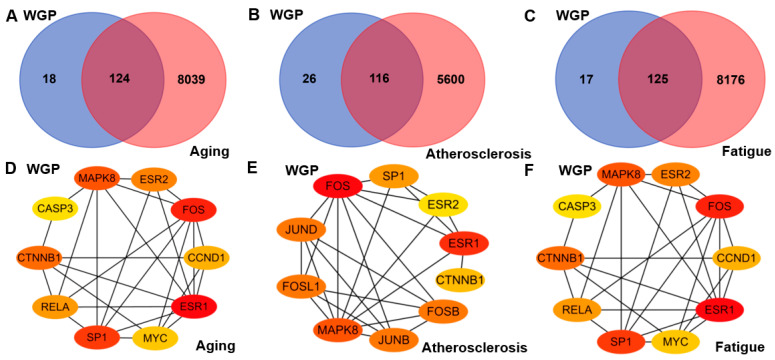
Network pharmacological analysis of WGP. (**A**–**C**) Venn diagram of the action targets of WGP and the targets of aging (**A**), atherosclerosis (**B**), and fatigue (**C**). (**D**–**F**) Protein-protein interaction network reflected in cytohubba-MCC identified hub genes in the gene lists for WGP-aging (**D**), WGP-atherosclerosis (**E**), and WGP-fatigue (**F**). Node size and color in the network graph are positively correlated with the degree value. Redder colors and larger nodes represent larger degree value.

The targets related to aging, atherosclerosis, and fatigue were obtained from four databases. They were 8163, 5716, and 8301, respectively. Venn diagram analysis identified 124, 116, and 125 intersection targets, which represent the overlapping targets between ones from WGP active ingredients and aging, atherosclerosis, or fatigue, respectively (Figure 11A–C).

#### 3.4.3. PPI Network and Screening of Key Targets

To further investigate the molecular mechanisms and functions of specific proteins, the STRING database was utilized to construct three PPI network graphs, namely WGP-aging, WGP-atherosclerosis, and WGP-fatigue (Figure S3). The results retrieved from STRING were imported into Cytoscape 3.9.1 for further analysis, and the top 10 core genes were filtered using the CytoHubba plugin (Figure 11D–F). Notably, the top 10 hub genes were the same for WGP-aging and WGP-fatigue. The top 10 hub genes of WGP-atherosclerosis were ranked from high to low according to their scores, which were FOS, ESR1, MAPK8, JUND, JUNB, FOSL1, FOSB, SP1, CTNNB1, and ESR2.

#### 3.4.4. GO Function and KEGG Pathway Enrichment Analysis

**Figure 12:**
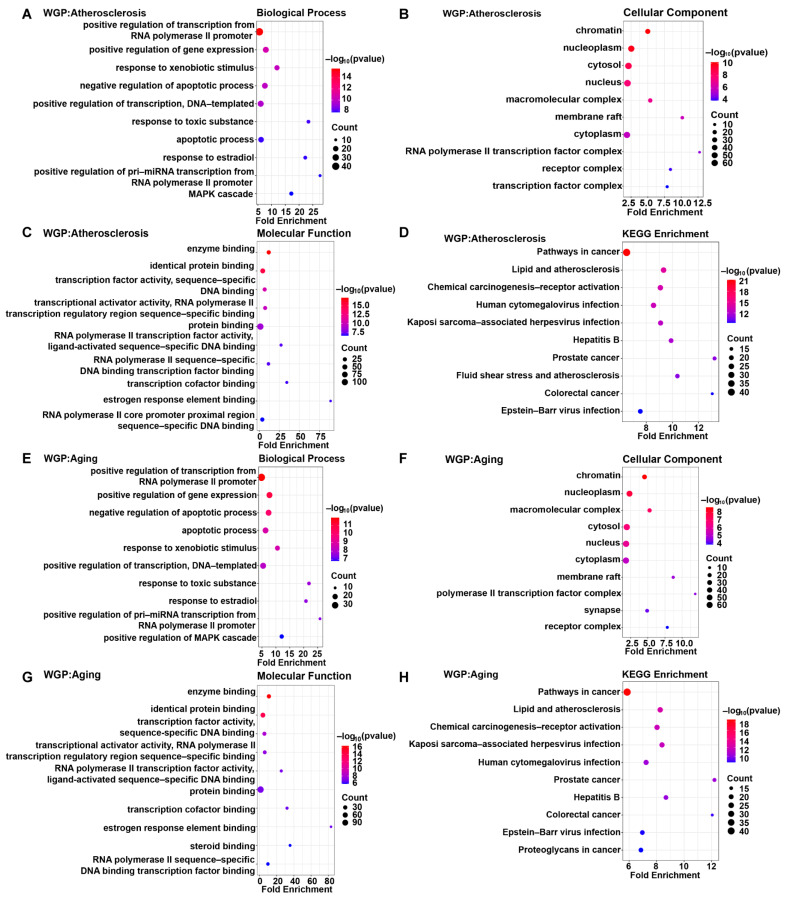
GO enrichment analysis and KEGG pathway analysis. (**A**–**D**) GO enrichment analysis and KEGG pathway of intersection target of WGP and atherosclerosis (FDR ≤ 0.05). The biological processes (top 10, (**A**)), the cellular components (top 10, (**B**)), the molecular functions (top 10, (**C**)), and the KEGG pathways (top 10, (**D**)). (**E**–**H**) GO enrichment analysis and KEGG pathway analysis for WGP-Aging (FDR ≤ 0.05). The biological processes (top 10, (**E**)), the cellular components (top 10, (**F**)), the molecular functions (top 10, (**G**)), and the KEGG pathways (top 10, (**H**)) analysis for WGP-aging. The bubble size represents the number of targets in the pathway. The bubble color indicates the magnitude of the −log_10_(*p*) values.

To explore the potential anti-atherosclerosis mechanism of WGP, GO function and KEGG pathway enrichment analyses were performed. There were 237 GO entries according to FDR < 0.05, including 156 BP terms, 30 CC terms, and 51 MF terms. An analysis of these data was performed using a bubble chart, which represented the ten most enhanced GO functions (Figure 12A–C) and each KEGG pathway (Figure 12D). In terms of BP, the target genes were mostly related to the positive regulation of transcription from RNA polymerase II promoter, the positive regulation of gene expression, and the response to exogenous stimuli, etc. In terms of CC, the nucleus was the biggest proportion base in the percentage of genes. MF was dominated by enzyme binding, identical-protein binding, transcription-factor activity, sequence-specific DNA binding, etc. In addition, a KEGG analysis revealed that the active ingredients of WGP might affect multiple pathways, including inflammatory pathways, lipids and atherosclerosis, and cancer-related pathways. We also performed GO and KEGG pathway-enrichment analyses for anti-aging (Figure 12E–H) and anti-fatigue (Figure S4), respectively. For anti-aging, the GO analysis yielded a total of 251 items (*p* < 0.05), with 153 items for BP, 36 for CC, and 62 for MF. The number of GO items for anti-fatigue was the same as that for anti-aging. We generated bubble plots for the top 10 GO entries. We performed KEGG pathway-enrichment analysis of WGP with targets of anti-aging and anti-fatigue effects, respectively (*p* < 0.05), with the same number of cellular pathways producing effects for anti-aging and anti-fatigue, both with a total of 119 identified pathways. They were mainly focused on inflammatory pathways, lipid-metabolism pathways, hormone-regulation pathways, cellular-senescence pathways, and cancer-related pathways.

#### 3.4.5. Molecular Docking

**Figure 13:**
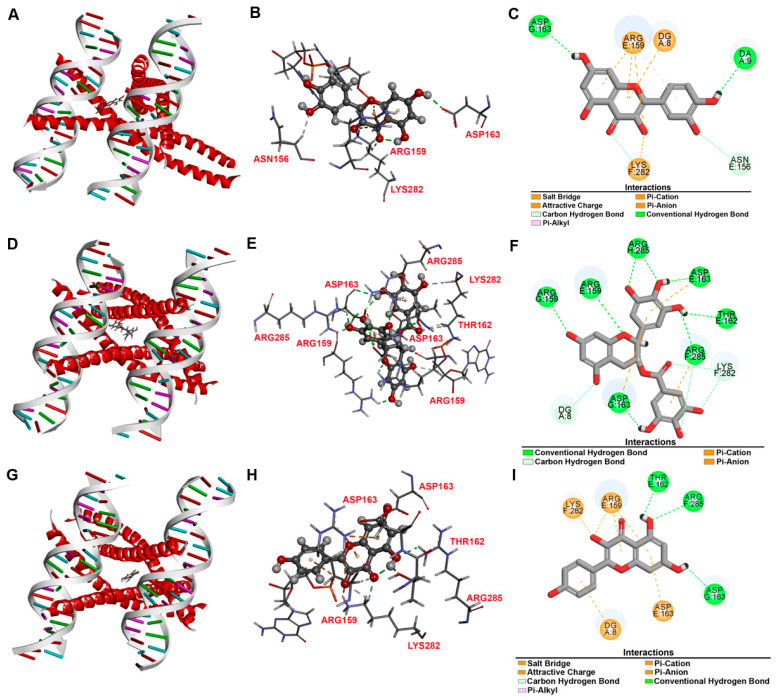
Molecular docking to model the interaction between FOS and three active ingredients of WGP (quercetin, EGCG, or kaempferol). (**A**–**C**) Interaction of FOS and quercetin. (**A**) Binding conformation of FOS-quercetin complex. (**B**,**C**) Electrical interactions of residue of FOS with quercetin. (**D**–**F**) Interaction of FOS and EGCG. (**D**) Binding conformation of FOS-EGCG complex. (**E**,**F**) Electrical interactions of residue on FOS with EGCG. (**G**–**I**) Interaction of FOS and kaempferol. (**G**) Binding conformation of FOS-kaempferol complex. (**H**,**I**) Electrical interactions of residue on FOS with kaempferol.

Based on the WGP compound-target-disease network, the top three major active ingredients were screened as quercetin, EGCG, and kaempferol. According to the topology analysis, the top three core genes of WGP-aging and WGP-fatigue were the same as ESR1, FOS, and SP1. The top three core genes of WGP-atherosclerosis were FOS, ESR1, and MAPK8. Therefore, the main active ingredients were molecularly docked with FOS, ESR1, MAPK8, and SP1, respectively. Binding energy values of less than −5 kcal/mol indicated strong binding between the ligand and the receptor [34]. The top three WGP ingredients (quercetin, EGCG, and kaempferol) could bind stably to the top four core genes of aging, atherosclerosis, and fatigue (FOS, ESR1, MAPK8, and SP1), except that EGCG could not dock successfully with SP1 (Figure 13A–I and Figures S5–S7). The molecular-binding energies of the top three WGP ingredients to MAPK8 were more stable than those of the other target genes (Table S3). In addition, the molecular-binding energies of the top three WGP ingredients to FOS were ranked from low to high as −57.10 kcal/mol (EGCG), −46.73 kcal/mol (kaempferol), and −46.16 kcal/mol (quercetin). It was suggested that QUR, EGCG, and kaempferol are more likely to exert their therapeutic effects through the above targets.

## 4. Discussion

WGP was prepared using patent technology with a slurry/gelatinization-dehydration/dryness–crush/screen process. The preparation process is energy-efficient, friendly to humans and the environment, without the addition of any chemical additives, and easy to scale up. The obtained WGP has good rehydration and fast dispersion in water. All the nutrients are preserved in the final products. Toxic ingredients such as 4′-O-methylpyridoxine can be removed by heating [22,35]. Active ingredients in ginkgo, such as flavonoids, terpene lactones, sugar, protein, and unsaturated fatty acids, may contribute to curative effects [36].

Aging is a serious situation that lowers one’s quality of life by impairing sensory and motor function and leading to irreversible organ injuries [37]. Decreased memory and cognitive function are common in aging and aging-related nervous system diseases [38]. The water maze test focused on evaluating the ability for spatial learning, non-spatial discrimination learning, and memory. It has been used as a powerful method to measure the cognition of mice [39]. Our results showed that the mice administered WGP increased exercise, as well as a higher time and distance in the targeted quadrant (Figure 1). They suggested that aging mice had poor learning and memory abilities, but an improvement was obtained after treatment with WGP and VE (a positive drug). Ginkgo biloba extract showed good efficacy in promoting episodic memory function in patients with mild cognitive impairment and impaired cognitive and memory abilities in Alzheimer’s disease [40]. The continuous administration of excess D-gal induced excessive ROS production and advanced glycation end products’ expression, finally leading to oxidative damage to the body [41]. The D-gal-induced aging model is suitable to imitate the aging process, during which oxidative stress and free radicals may be involved [42]. Our study showed that the effect of WGP on delaying aging and abated memory might be attributed to antioxidant and immune function (Figure 4). SOD is one of the important components of antioxidant enzyme systems for scavenging excessive free radicals [43]. The increase in MDA is a natural product of lipid oxidation and one of the obvious oxidative stress characteristics [44,45]. MAO is responsible for the oxidative deamination of various biogenic and xenobiotic amines. MAO increases with age [46]. WGP played a key role in improving SOD and reducing MDA and MAO, which led to regulated oxidative stress. The spleen and thymus are important immune organs [47,48]. Their structures and functions are related to immunity and degeneration (Figure 3). They decrease with age. The deterioration of the immune function is the main cause of aging. The increase in the spleen and thymus indexes contributes to the prevention of aging. There were no statistically significant differences among the heart, liver, lung, kidney, or brain indexes of the controls and/or WGP-treated groups (Figure S1), suggesting that no obvious organ damage occurred in aging mice or was induced via WGP [49]. Similarly, the improvement in pathological changes in organs is conducive to maintaining body function (Figure 5) [50,51].

Atherosclerosis is the underlying cause of CVDs, and hyperlipidemia is one of the major risk factors for increasing the occurrence of atherosclerosis and CVDs [52]. Hyperlipidemia is characterized by enhanced serum levels of TG, TC, and LDL-C and a reduced positive indicator of a high serum HDL-C level [53]. Therefore, functional materials are useful for preventing atherosclerosis and CVDs by regulating lipid metabolism and disturbances of hyperlipidemia [54]. Ginkgo biloba leaves and their extracts have been widely applied to treat CVDs and atherosclerosis, and the mechanism may involve decreased inflammatory factor levels in serum, improved coronary artery circulation, and decreased membrane damage caused by free radicals [55,56,57]. Flavonoids and terpene trilactones are active ingredients in ginkgo. They are potential compounds for preventing atherosclerosis and related diseases [36,58]. Here, our study concluded that WGP had a hypolipidemic effect by adjusting the increase in LDL-C and TG, as well as promoting the level of HDL-C, similar to other anti-atherosclerosis ingredients (Figure 5 and Figure 6) [59]. We further confirmed that WGP prevented atherosclerosis and related CVDs by inhibiting the occurrence of endothelial damage and atheromatous plaque in the initial step of atherosclerosis [60].

Exercise performance and energy metabolism are indicators reflecting the body’s ability to relieve fatigue. Forced swimming and climbing tests are suitable for evaluating exercise performance [61]. WGP significantly increased the forced swimming time and climbing time of mice (Figure 7), demonstrating the obvious antifatigue effect by improving exercise performance. We further determined the metabolic product content to determine whether WGP had an anti-fatigue effect (Figure 8). Aerobic metabolism cannot meet energy requirements and anaerobic glycolysis is conducted during high-intensity exercise [62]. BLA and BUN are considered two parameters to evaluate fatigue. BLA is the glycolysis product of carbohydrates under anaerobic conditions, and its accumulation may cause metabolic dysfunction and fatigue [63,64]. Prolonged strenuous exercise caused the body to accelerate glycolysis, reduce glycogen storage, and produce lactic acid. The increased BLA level brought about a reduction in pH in muscle tissue and blood and caused acidosis, finally leading to the production of fatigue. The reduced BLA showed an anti-fatigue effect. Treatment with WGP, especially at a high dose, markedly reduced the overproduction and accumulation of BLA. BUN is the metabolic product of protein and an amino acid when energy from sugar or fat metabolism cannot meet needs. The accumulation of BUN leads to a decrease in muscle contraction strength and induces fatigue [65]. WGP had a small impact on BUN. The body’s energy supply and its hepatic and muscle glycogen stores maintain homeostasis to achieve exercise sustainability and body recovery [66]. We confirmed that the LG and MG content in mice treated with WGP-H significantly increased, suggesting that WGP provided the mice with sufficient energy storage for exercise.

We identified 270 common effect targets of the 15 bioactive compounds in WGP that are associated with aging, atherosclerosis, or fatigue. These targets may be crucial for the treatment of aging, atherosclerosis, or fatigue (Table S2). The intersection targets of WGP with aging, atherosclerosis, or fatigue were analyzed using Venn diagrams and found to be 124, 116, and 125 targets, respectively (Figure 9A–C). Biological functions cannot occur autonomously from individual proteins, but they are generated via complex networks of proteins [67]. Ten hub genes were identified through the protein-interaction network analysis of active ingredients with three common disease targets, respectively. FOS, ESR1, MAPK8, and SP1 are important targets for anti-aging, anti-atherosclerosis, and anti-fatigue. FOS can regulate the production of vascular endothelial growth factor and be involved in the biosynthesis of cholesterol (a key regulator of lipid metabolism) [68]. The FOS/MAPK signaling pathway inhibits M1 macrophage polarization and promotes M2 polarization, inhibiting and stabilizing the progression of atherosclerosis plaques [69]. Matrix metalloproteinase-1 (MMP-1) is the major protease responsible for collagen breakage, and it accelerates aging production. The phosphorylation of c-FOS (a gene product of FOS) can reduce ROS production, downregulating MMP-1 mRNA protein expression and delaying aging [70]. ESR1 activates specific target genes in vascular smooth muscle, inhibits smooth-muscle-cell migration, and it accelerates endothelial cell growth to produce atherosclerosis-protective effects [71]. The downregulation of the expression of MAPK8 can provide therapeutic atherosclerosis effects by promoting endothelial cell proliferation, inhibiting apoptosis, and suppressing the inflammatory response [72]. SP1 is an upstream transcription factor of proline/serine-rich coiled-coil protein 1 (PSRC1), and it represses PSRC1 transcription. The overexpression of PSRC1 reduces the macrophage inflammatory response and delays the development of atherosclerosis [73]. Sp1 is an anti-aging transcription factor in telomere uncapping-induced senescence. The downregulation of Sp1 leads to senescence through downregulating nuclear translocation [74]. Meanwhile, we molecularly docked the main active components of WGP with FOS, ESR1, MAPK8, and SP1. The molecular-binding energy results indicated that the bond between active components and target genes was stable. The molecular mechanism analysis revealed that the active components of WGP have a positive influence on the four potentially important therapeutic targets of aging, atherosclerosis, or fatigue (i.e., FOS, ESR1, MAPK8, and SP1). The GO and KEGG pathway enrichment analyses were performed to analyze the common targets of WGP with anti-aging, anti-atherosclerosis, and anti-fatigue. Among the major hub genes FOS, ESR1, MAPK8, and SP1 were 44, 8, 64, and 10 enriched KEGG cellular pathways, respectively. In the anti-atherosclerosis KEGG cell pathway analysis, we identified important signaling pathways mostly related to anti-inflammation. The common anti-inflammatory signaling pathways mainly focused on the TNF signaling pathway, IL-17 signaling pathway, MAPK signaling pathway, NF-kappa B signaling pathway, Toll-like receptor signaling pathway, and JAK-STAT signaling pathway. Previous studies have reported that the above anti-inflammatory pathways slow down the progression of atherosclerosis by inhibiting the production of inflammatory factors [75,76]. Among the anti-aging KEGG cell pathway molecules, WGP mainly exerts anti-aging effects through anti-inflammatory (such as the NF-kappa B signaling pathway) and metabolic (such as the AMPK signaling pathway) pathways. The molecular mechanism of anti-aging is related to the regulation of the SIRT1/NF-κB pathway [77]. The activation of the AMPK signaling pathway can improve the quality and function of skeletal muscle and achieve anti-aging effects [78]. Among the anti-fatigue KEGG cell pathway molecules, WGP mainly exerts anti-fatigue effects through cellular senescence, apoptosis, and other pathways.

Our findings confirmed the health benefits of WGP and discovered multiple potential targets and pathways. However, more research, such as a pharmacokinetic study and component distribution, is needed to fully elucidate the in vivo process and mechanisms of action.

## 5. Conclusions

WGP has the multifunctional efficacy of anti-aging, anti-atherosclerosis, and anti-fatigue. The underlying mechanisms can be illustrated from the multi-component, multi-target, and multi-pathway perspectives through an experimental evaluation and a network pharmacology analysis. WGP exhibited an anti-aging effect on D-Gal-induced mice. This effect was ascribed to memory improvement, an antioxidant capacity increase, and organ function maintenance. WGP reduced atherosclerosis progression in HFD-fed rats by adjusting lipid disorders and inhibiting endothelial damage in arteries. WGP exhibited the ability to enhance exercise performance, regulate the production or accumulation of metabolic products, and increase the glycogen content to relieve fatigue. In summary, WGP has multifunctional efficacies against aging, atherosclerosis, and fatigue. Here, we identified 15 potential compounds and 124, 116, and 125 targets related to anti-aging, anti-atherosclerosis, and anti-fatigue, respectively. Quercetin, EGCG and kaempferol were found to be the main active components of WGP. Moreover, FOS, ESR1, MAPK8, and SP1 were distinguished as hub gene targets via a PPI network analysis. The above three active components of WGP can stably bind to the four hub gene targets via a molecular-docking simulation analysis.

## Data Availability

All data and materials used are available in the manuscript and Supplementary Materials.

## Supplementary Materials

The following supporting information can be downloaded at https://www.mdpi.com/article/10.3390/antiox13091104/s1: Table S1: Twenty-one bioactive compounds meeting screening criteria from TCMSP; Table S2: Target names converted into symbols of corresponding genes using UniProt database; Table S3: The negative CDOCKER interaction energy of the bioactive compounds to the core gene targets; Figure S1: Effects of WGP on organ indexes; Figure S2: Effects of WGP on serum biochemical lipid parameter LDH.; Figure S3: PPI network of WGP for anti-aging, anti-atherosclerosis, and anti-fatigue using STRING database; Figure S4: GO enrichment analysis and KEGG pathway analysis for WGP–Fatigue; Figure S5: Molecular docking to model the interaction between ESR1 and three active ingredients of WGP; Figure S6: Molecular docking to model the interaction between MAPK8 and three active ingredients of WGP; and Figure S7: Molecular docking to model the interaction between SP1 and two active ingredients of WGP (quercetin or kaempferol).

## Abbreviations

**Abbreviation**	**Full** **name**
AI	atherosclerosis index
BLA	blood lactic acid
CVDs	cardiovascular diseases
D-gal	D-galactose
GB	Ginkgo biloba L
HDL-C	high-density lipoprotein cholesterol
LDL-C	low-density lipoprotein cholesterol
LG	liver glycogen
MAO	monoamine oxidase
MDA	malondialdehyde
MG	muscle glycogen
TC	total cholesterol
TG	triglyceride
WGP	whole ginkgo biloba L powder
TCMSP	traditional Chinese medicine systems pharmacology database and analysis platform
OB	oral bioavailability
DL	drug likeness
OMIM	online mendelian inheritance in man
TTD database	therapeutic target database
PPI	protein–protein interaction
GO	gene ontology
KEGG	Kyoto encyclopedia of genes and genomes
FDR	false discovery rate
BP	biological process
CC	cellular component
MF	molecular function
EGCG	(-)-epigallocatechin-3-gallate

## References

[1] López-Otín C., Blasco M.A., Partridge L., Serrano M., Kroemer G. (2023). Hallmarks of aging: An expanding universe. Cell.

[2] Guo J., Huang X., Dou L., Yan M., Shen T., Tang W., Li J. (2022). Aging and aging-related diseases: From molecular mechanisms to interventions and treatments. Signal Transduct. Target. Ther..

[3] Partridge L., Fuentealba M., Kennedy B.K. (2020). The quest to slow ageing through drug discovery. Nat. Rev. Drug Discov..

[4] Li D., Ma J., Wei B., Gao S., Lang Y., Wan X. (2023). Effectiveness and safety of ginkgo biloba preparations in the treatment of Alzheimer’s disease: A systematic review and meta-analysis. Front. Aging Neurosci..

[5] Libby P. (2021). The changing landscape of atherosclerosis. Nature.

[6] Velayutham N., Lee R.T. (2023). AGrimlink: The association between subclinical atherosclerosis and epigenetic age. Eur. Heart J..

[7] Li X., Lu L., Chen J., Zhang C., Chen H., Huang H. (2020). New insight into the mechanisms of ginkgo biloba extract in vascular aging prevention. Curr. Vasc. Pharmacol..

[8] Björkegren J.L.M., Lusis A.J. (2022). Atherosclerosis: Recent developments. Cell.

[9] Davies K., Dures E., Ng W.-F. (2021). Fatigue in inflammatory rheumatic diseases: Current knowledge and areas for future research. Nat. Rev. Rheumatol..

[10] Fabi A., Bhargava R., Fatigoni S., Guglielmo M., Horneber M., Roila F., Weis J., Jordan K., Ripamonti C.I. (2020). Cancer-related fatigue: ESMO Clinical Practice Guidelines for diagnosis and treatment. Ann. Oncol..

[11] Zhang Z., Zhang X., Wang X., Guo X., Yan X., Li Z., Li W. (2024). Chemical constituents, pharmacological activities and quality evaluation methods of genus Hippocampus: A comprehensive review. Chin. Herb. Med..

[12] Liu H., Wang X., Wang G., Cui P., Wu S., Ai C., Hu N., Li A., He B., Shao X. (2021). The nearly complete genome of Ginkgo biloba illuminates gymnosperm evolution. Nat. Plants.

[13] Li Y., Sheng Y., Liu J., Xu G., Yu W., Cui Q., Lu X., Du P., An L. (2022). Hair-growth promoting effect and anti-inflammatory mechanism of Ginkgo biloba polysaccharides. Carbohydr. Polym..

[14] Christen Y., Maixent J.M. (2002). What is Ginkgo biloba extract EGb 761? An overview—From molecular biology to clinical medicine. Cell. Mol. Biol..

[15] Hui W., Huang W., Zheng Z., Li Y., Li P., Yang H. (2023). Ginkgo biloba extract promotes Treg differentiation to ameliorate ischemic stroke via inhibition of HIF-1alpha/HK2 pathway. Phytother. Res..

[16] Wang Y., Xu Y., Xu X., Wang H., Wang D., Yan W., Zhu J., Hao H., Wang G., Cao L. (2022). Ginkgo biloba extract ameliorates atherosclerosis via rebalancing gut flora and microbial metabolism. Phytother. Res..

[17] Xie L., Zhu Q., Lu J. (2022). Can we use Ginkgo biloba extract to treat Alzheimer’s disease? Lessons from preclinical and clinical Studies. Cells.

[18] Barbalho S.M., Direito R., Laurindo L.F., Marton L.T., Guiguer E.L., Goulart R.A., Tofano R.J., Carvalho A.C.A., Flato U.A.P., Capelluppi Tofano V.A. (2022). Ginkgo biloba in the aging process: A narrative review. Antioxidants.

[19] Zuo W., Yan F., Zhang B., Li J., Mei D. (2017). Advances in the studies of Ginkgo biloba leaves extract on aging-related diseases. Aging Dis..

[20] Wang H., Shi M., Cao F., Su E. (2022). Ginkgo biloba seed exocarp: A waste resource with abundant active substances and other components for potential applications. Food Res. Int..

[21] Wang X., Deng Y., Xie P., Liu L., Zhang C., Cheng J., Zhang Y., Liu Y., Huang L., Jiang J. (2023). Novel bioactive peptides from ginkgo biloba seed protein and evaluation of their alpha-glucosidase inhibition activity. Food Chem..

[22] Boateng I.D. (2022). A critical review of current technologies used to reduce ginkgotoxin, ginkgotoxin-5’-glucoside, ginkgolic acid, allergic glycoprotein, and cyanide in *Ginkgo biloba* L. seed. Food Chem..

[23] Nogales C., Mamdouh Z.M., List M., Kiel C., Casas A.I., Schmidt H. (2022). Network pharmacology: Curing causal mechanisms instead of treating symptoms. Trends Pharmacol. Sci..

[24] Lee H., Wang Z., Deng Z., Wang Y. (2024). Assessment of Six Blackberry Cultivars Using a Combination of Metabolomics, Biological Activity, and Network Pharmacology Approaches. Antioxidants.

[25] Nam D.G., Kim M., Choi A.J., Choe J.S. (2024). Health Benefits of Antioxidant Bioactive Compounds in Ginger (*Zingiber officinale*) Leaves by Network Pharmacology Analysis Combined with Experimental Validation. Antioxidants.

[26] Jin H., Yang C., Jiang C., Li L., Pan M., Li D., Han X., Ding J. (2022). Evaluation of Neurotoxicity in BALB/c Mice following Chronic Exposure to Polystyrene Microplastics. Environ. Health Perspect..

[27] Zhang C.J., Guo J.Y., Cheng H., Li L., Liu Y., Shi Y., Xu J., Yu H.T. (2020). Spatial structure and anti-fatigue of polysaccharide from Inonotus obliquus. Int. J. Biol. Macromol..

[28] Cai M., Zhu H., Xu L., Wang J., Xu J., Li Z., Yang K., Wu J., Sun P. (2023). Structure, anti-fatigue activity and regulation on gut microflora in vivo of ethanol-fractional polysaccharides from Dendrobium officinale. Int. J. Biol. Macromol..

[29] Chen J., Wu X., Yu R. (2023). Unraveling the Therapeutic Mechanism of Saussurea involucrata against Rheumatoid Arthritis: A Network Pharmacology and Molecular Modeling-Based Investigation. Nutrients.

[30] Liu Y., Hou M., Pan Z., Tian X., Zhao Z., Liu T., Yang H., Shi Q., Chen X., Zhang Y. (2022). Arctiin-reinforced antioxidant microcarrier antagonizes osteoarthritis progression. J. Nanobiotechnology.

[31] Chen S., Li B., Chen L., Jiang H. (2023). Uncovering the mechanism of resveratrol in the treatment of diabetic kidney disease based on network pharmacology, molecular docking, and experimental validation. J. Transl. Med..

[32] Shen M., Duan C., Xie C., Wang H., Li Z., Li B., Wang T. (2022). Identification of key interferon-stimulated genes for indicating the condition of patients with systemic lupus erythematosus. Front. Immunol..

[33] Wang M., Gong L., Luo Y., He S., Zhang X., Xie X., Li X., Feng X. (2023). Transcriptomic analysis of asthma and allergic rhinitis reveals CST1 as a biomarker of unified airways. Front. Immunol..

[34] Chen Z., Zhang S., Sun X., Meng D., Lai C., Zhang M., Wang P., Huang X., Gao X. (2024). Analysis of the Protective Effects of Rosa roxburghii-Fermented Juice on Lipopolysaccharide-Induced Acute Lung Injury in Mice through Network Pharmacology and Metabolomics. Nutrients.

[35] Lim H.B., Kim D.H. (2018). Effect of heat treatment on 4’-O-methylpyridoxine (MPN) content in Ginkgo biloba seed extract solution. J. Sci. Food Agric..

[36] Liu X.G., Lu X., Gao W., Li P., Yang H. (2022). Structure, synthesis, biosynthesis, and activity of the characteristic compounds from *Ginkgo biloba* L.. Nat. Prod. Rep..

[37] Lorenzo E.C., Kuchel G.A., Kuo C.L., Moffitt T.E., Diniz B.S. (2023). Major depression and the biological hallmarks of aging. Ageing Res. Rev..

[38] Hofer S.J., Liang Y., Zimmermann A., Schroeder S., Dengjel J., Kroemer G., Eisenberg T., Sigrist S.J., Madeo F. (2021). Spermidine-induced hypusination preserves mitochondrial and cognitive function during aging. Autophagy.

[39] Hong Y., Liu Q., Peng M., Bai M., Li J., Sun R., Guo H., Xu P., Xie Y., Li Y. (2020). High-frequency repetitive transcranial magnetic stimulation improves functional recovery by inhibiting neurotoxic polarization of astrocytes in ischemic rats. J. Neuroinflammation.

[40] Zhang L.D., Ma L., Zhang L., Dai J.G., Chang L.G., Huang P.L., Tian X.Q. (2015). Hyperbaric Oxygen and Ginkgo Biloba Extract Ameliorate Cognitive and Memory Impairment via Nuclear Factor Kappa-B Pathway in Rat Model of Alzheimer’s Disease. Chin. Med. J..

[41] Xie G., Xu Z., Li F., Kong M., Wang P., Shao Y. (2024). Aerobic Exercise Ameliorates Cognitive Disorder and Declined Oxidative Stress via Modulating the Nrf2 Signaling Pathway in D-galactose Induced Aging Mouse Model. Neurochem. Res..

[42] Wang Y., Qiu H., Chen S., Li D., Zhao X., Guo M., Li N., Chen C., Qin M., Zhou Y. (2024). MicroRNA-7 deficiency ameliorates d-galactose-induced aging in mice by regulating senescence of Kupffer cells. Aging Cell.

[43] Sun Y., Liu X., Wang L., Xu L., Liu K., Xu L., Shi F., Zhang Y., Gu N., Xiong F. (2022). High-performance SOD mimetic enzyme Au@Ce for arresting cell cycle and proliferation of acute myeloid leukemia. Bioact. Mater..

[44] Liu Q., Sun Y.M., Huang H., Chen C., Wan J., Ma L.H., Sun Y.Y., Miao H.H., Wu Y.Q. (2021). Sirtuin 3 protects against anesthesia/surgery-induced cognitive decline in aged mice by suppressing hippocampal neuroinflammation. J. Neuroinflamm..

[45] Chen R., Yang J., Wu M., Zhao D., Yuan Z., Zeng L., Hu J., Zhang X., Wang T., Xu J. (2023). M2 Macrophage Hybrid Membrane-Camouflaged Targeted Biomimetic Nanosomes to Reprogram Inflammatory Microenvironment for Enhanced Enzyme-Thermo-Immunotherapy. Adv. Mater..

[46] Valsecchi V., Errico F., Bassareo V., Marino C., Nuzzo T., Brancaccio P., Laudati G., Casamassa A., Grimaldi M., D’Amico A. (2023). SMN deficiency perturbs monoamine neurotransmitter metabolism in spinal muscular atrophy. Commun. Biol..

[47] Li W., Hu X., Wang S., Jiao Z., Sun T., Liu T., Song K. (2020). Characterization and anti-tumor bioactivity of astragalus polysaccharides by immunomodulation. Int. J. Biol. Macromol..

[48] Chen Y., Zhang M., Zhao H., Liu Y., Wang T., Lei T., Xiang X., Lu L., Yuan Z., Xu J. (2022). Oral supramolecular nanovectors for dual natural medicine codelivery to prevent gastric mucosal lesion. Nanoscale.

[49] Xie X., Li Y., Zhao D., Fang C., He D., Yang Q., Yang L., Chen R., Tan Q., Zhang J. (2020). Oral administration of natural polyphenol-loaded natural polysaccharide-cloaked lipidic nanocarriers to improve efficacy against small-cell lung cancer. Nanomedicine.

[50] Jebari K., Charradi K., Mahmoudi M., Kadri S., Ben-Attia M., Mousslim M., El May M.V., Limam F., Aouani E. (2022). Grape Seed Flour Extends Longevity by Improving Multi-Organ Dysfunction and Age-Associated Oxidative Stress and Inflammation in Healthy Rat. J. Gerontol. A Biol. Sci. Med. Sci..

[51] Zhao J., Li Y., He D., Hu X., Li K., Yang Q., Fang C., Zhong C., Yang J., Tan Q. (2020). Natural Oral Anticancer Medication in Small Ethanol Nanosomes Coated with a Natural Alkaline Polysaccharide. ACS Appl. Mater. Interfaces.

[52] Simha V. (2020). Management of hypertriglyceridemia. BMJ.

[53] Ridker P.M., Bhatt D.L., Pradhan A.D., Glynn R.J., MacFadyen J.G., Nissen S.E., Prominent R.-I., Investigators S. (2023). Inflammation and cholesterol as predictors of cardiovascular events among patients receiving statin therapy: A collaborative analysis of three randomised trials. Lancet.

[54] Bao X., Liang Y., Chang H., Cai T., Feng B., Gordon K., Zhu Y., Shi H., He Y., Xie L. (2024). Targeting proprotein convertase subtilisin/kexin type 9 (PCSK9): From bench to bedside. Signal Transduct. Target. Ther..

[55] Asiwe J.N., Ojetola A.A., Ekene N.E., Osirim E., Nnamudi A.C., Oritsemuelebi B., Onuelu J.E., Asiwe N., Eruotor H.O., Inegbenehi S. (2024). Pleiotropic attenuating effect of Ginkgo biloba against isoprenaline-induced myocardial infarction via improving Bcl-2/mTOR/ERK1/2/Na(+), K(+)-ATPase activities. Chin. Herb. Med..

[56] Wang L.T., Huang H., Chang Y.H., Wang Y.Q., Wang J.D., Cai Z.H., Efferth T., Fu Y.J. (2022). Biflavonoids from Ginkgo biloba leaves as a novel anti-atherosclerotic candidate: Inhibition potency and mechanistic analysis. Phytomedicine.

[57] Pierre S.V., Lesnik P., Moreau M., Bonello L., Droy-Lefaix M.T., Sennoune S., Duran M.J., Pressley T.A., Sampol J., Chapman J. (2008). The standardized Ginkgo biloba extract Egb-761 protects vascular endothelium exposed to oxidized low density lipoproteins. Cell. Mol. Biol..

[58] Wang Q., Zhao X., Jiang Y., Jin B., Wang L. (2023). Functions of Representative Terpenoids and Their Biosynthesis Mechanisms in Medicinal Plants. Biomolecules.

[59] Choghakhori R., Abbasnezhad A., Yazdi M., Ahmadvand H. (2023). Antidiabetic, hypolipidemic, and antioxidant activities of Pistacia atlantica: A systematic review and meta-analysis of preclinical studies. Phytother. Res..

[60] Li Y., Shi G., Liang W., Shang H., Li H., Han Y., Zhao W., Bai L., Qin C. (2023). Allogeneic Adipose-Derived Mesenchymal Stem Cell Transplantation Alleviates Atherosclerotic Plaque by Inhibiting Ox-LDL Uptake, Inflammatory Reaction and Endothelial Damage in Rabbits. Cells.

[61] Guan T., Li S., Guan Q., Shi J.S., Lu Z.M., Xu Z.H., Geng Y. (2022). Spore Powder of Paecilomyces hepiali Shapes Gut Microbiota to Relieve Exercise-Induced Fatigue in Mice. Nutrients.

[62] Fountain W.A., Bopp T.S., Bene M., Walston J.D. (2024). Metabolic dysfunction and the development of physical frailty: An aging war of attrition. Geroscience.

[63] Kamel K.S., Oh M.S., Halperin M.L. (2020). L-lactic acidosis: Pathophysiology, classification, and causes; emphasis on biochemical and metabolic basis. Kidney Int..

[64] Gu J., Huang Y., Yan Z., He D., Zhang Y., Xu J., Li Y., Xie X., Xie J., Shi D. (2020). Biomimetic Membrane-Structured Nanovesicles Carrying a Supramolecular Enzyme to Cure Lung Cancer. ACS Appl. Mater. Interfaces.

[65] Jiang P., Ji X., Xia J., Xu M., Hao F., Tong H., Jiao L. (2023). Structure and potential anti-fatigue mechanism of polysaccharides from Bupleurum chinense DC. Carbohydr. Polym..

[66] Hargreaves M., Spriet L.L. (2020). Skeletal muscle energy metabolism during exercise. Nat. Metab..

[67] Xu J., Wu M., Yang J., Zhao D., He D., Liu Y., Yan X., Liu Y., Pu D., Tan Q. (2024). Multimodal smart systems reprogramme macrophages and remove urate to treat gouty arthritis. Nat. Nanotechnol..

[68] Choi Y., Jeon H., Akin J.W., Curry T.E., Jo M. (2021). The FOS/AP-1 Regulates Metabolic Changes and Cholesterol Synthesis in Human Periovulatory Granulosa Cells. Endocrinology.

[69] Jin Z., Li J., Pi J., Chu Q., Wei W., Du Z., Qing L., Zhao X., Wu W. (2020). Geniposide alleviates atherosclerosis by regulating macrophage polarization via the FOS/MAPK signaling pathway. Biomed. Pharmacother..

[70] Wu Y.Z., Tsai Y.Y., Chang L.S., Chen Y.J. (2021). Evaluation of Gallic Acid-Coated Gold Nanoparticles as an Anti-Aging Ingredient. Pharmaceuticals.

[71] Abulizi A., Simayi J., Nuermaimaiti M., Han M., Hailati S., Talihati Z., Maihemuti N., Nuer M., Khan N., Abudurousuli K. (2023). Quince extract resists atherosclerosis in rats by down-regulating the EGFR/PI3K/Akt/GSK-3beta pathway. Biomed. Pharmacother..

[72] Wang Y., Jia Q., Zhang Y., Wei J., Liu P. (2020). Amygdalin Attenuates Atherosclerosis and Plays an Anti-Inflammatory Role in ApoE Knock-Out Mice and Bone Marrow-Derived Macrophages. Front. Pharmacol..

[73] Pan H., Guo Z., Lv P., Hu K., Wu T., Lin Z., Xue Y., Zhang Y., Guo Z. (2023). Proline/serine-rich coiled-coil protein 1 inhibits macrophage inflammation and delays atherosclerotic progression by binding to Annexin A2. Clin. Transl. Med..

[74] Park J.H., Ryu S.J., Kim B.J., Cho H.J., Park C.H., Choi H.J.C., Jang E.J., Yang E.J., Hwang J.A., Woo S.H. (2021). Disruption of nucleocytoplasmic trafficking as a cellular senescence driver. Exp. Mol. Med..

[75] An H.J., Gwon M.G., Gu H., Bae S., Leem J., Lee J.B., Park K.K. (2023). STAT3/NF-kappaB decoy oligodeoxynucleotides inhibit atherosclerosis through regulation of the STAT/NF-kappaB signaling pathway in a mouse model of atherosclerosis. Int. J. Mol. Med..

[76] Huang R., Sun Y., Liu R., Zhu B., Zhang H., Wu H. (2024). ZeXieYin formula alleviates atherosclerosis by inhibiting the MAPK/NF-kappaB signaling pathway in APOE-/- mice to attenuate vascular inflammation and increase plaque stability. J. Ethnopharmacol..

[77] Xu C., Huang X., Tong Y., Feng X., Wang Y., Wang C., Jiang Y. (2020). Icariin modulates the sirtuin/NF-kappaB pathway and exerts anti-aging effects in human lung fibroblasts. Mol. Med. Rep..

[78] Nie L., He K., Qiu C., Li Q., Xiong B., Gao C., Zhang X., Jing M., Wu W., Liu J. (2024). Tetramethylpyrazine Nitrone alleviates D-galactose-induced murine skeletal muscle aging and motor deficits by activating the AMPK signaling pathway. Biomed. Pharmacother..

