# Supplementary material for: Ginkgo Biloba Bioactive Phytochemicals against Age-Related Diseases: Evidence from a Stepwise, High-Throughput Research Platform
Source: Antioxidants (Basel). 2024 Sep 12;13(9):1104. doi: 10.3390/antiox13091104 (PMC11429439; doi:10.3390/antiox13091104)
Supplement: Supplementary file 1 [file antioxidants-13-01104-s001.zip › antioxidants-3154877-supplementary.pdf]

Supplementary Materials

Ginkgo Biloba Bioactive Phytochemicals against Age-related Diseases: Evidence from A Stepwise High Throughput Research Platform

Yuming Yuan [1,†], Xiaoyan Xiang [1,†], Xuejun Jiang [1,†], Yingju Liu [1,†], Ming Zhang [2], Luyang Lu [3], Xinping Zhang [1], Xinyi Liu[1], Qunyou Tan [2,*] and Jingqing Zhang [1,*]

1 College of Pharmacy, Chongqing Medical University, Chongqing 400016, China; yuanyuming@stu.cqmu.edu.cn (Y.Y.); xiangxiaoyan0709@163.com (X.X.); angueun@126.com (X.J.); liuyingju@cqmu.edu.cn (Y.L.); xpzhang0223@163.com (X.Z.); 18398800121@163.com (X.L.)

2 Department of Thoracic Surgery, University-Town Hospital of Chongqing Medical University, Chongqing 401331, China; zhangming@hospital.cqmu.edu.cn (M.Z.)

3 College of Pharmacy, Southwest Minzu University, Chengdu 610041, China; luluyang@swun.edu.cn

* Correspondence: tanqunyou@hospital.cqmu.edu.cn (Q.T.); 13308300303@cqmu.edu.cn (J.Z.)

† Joint first authors.

Supplementary Materials Catalog

Supplementary Tables
Supplementary Figures

Supplementary Tables

Table S1. Bioactive compounds meeting screening criteria from TCMSP.

Table S2. Target names converted into symbols of corresponding genes using UniProt database.

Table S3. The negative CDOCKER interaction energy of the bioactive compounds to the core gene targets.

Table S1. Bioactive compounds meeting screening criteria from TCMSP.

MOL ID	Molecule Name	MW	OB≥30%	DL≥0.18
MOL011042	18alpha-hydroglycyrrhetic acid	488.73	38.93	0.71
MOL011059	ginkgolid B	424.44	42.84	0.73
MOL011061	ginkgolide B	424.44	46.14	0.73
MOL011063	heneicosadienoic acid	322.59	35.61	0.23
MOL011072	quinicine	324.46	75.44	0.33
MOL011073	scillaren A	692.88	56.12	0.21
MOL011074	scillaren A_qt	384.56	57.67	0.78
MOL011075	shikodonin	362.46	78.16	0.56
MOL001771	poriferast-5-en-3beta-ol	414.79	36.91	0.75
MOL002773	beta-carotene	536.96	37.18	0.58
MOL000449	stigmasterol	412.77	43.83	0.76
MOL000354	isorhamnetin	316.28	49.6	0.31
MOL000358	beta-sitosterol	414.79	36.91	0.75
MOL000392	formononetin	268.28	69.67	0.21
MOL000422	kaempferol	286.25	41.88	0.24
MOL004350	ruvoside_qt	390.57	36.12	0.76
MOL000492	(+)-catechin	290.29	54.83	0.24
MOL005236	gibberellin	346.41	81.59	0.53
MOL006821	(-)-epigallocatechin-3-gallate	458.4	55.09	0.77
MOL008691	alpha-carotene/ beta, epsilon-carotene	536.96	34.51	0.58
MOL000098	quercetin	302.25	46.43	0.28

Table S2. Target names converted into symbols of corresponding genes using UniProt database.

MOLID	Bioactive components	Target name	Genesymbol
MOL011072	Quinicine	Prostaglandin G/H synthase 1	PTGS1
MOL011072	Quinicine	Muscarinic acetylcholine receptor M3	CHRM3
MOL011072	Quinicine	Muscarinic acetylcholine receptor M1	CHRM1
MOL011072	Quinicine	Muscarinic acetylcholine receptor M5	CHRM5
MOL011072	Quinicine	Muscarinic acetylcholine receptor M4	CHRM4
MOL011072	Quinicine	Alpha-1A adrenergic receptor	ADRA1A
MOL011072	Quinicine	Gamma-aminobutyric acid receptor subunit alpha-1	GABRA1
MOL011074	Scillaren A_qt	Progesterone receptor	PGR
MOL011074	Scillaren A_qt	Mineralocorticoid receptor	NR3C2
MOL011074	Scillaren A_qt	Glucocorticoid receptor	NR3C1
MOL011074	Scillaren A_qt	Nuclear receptor coactivator 1	NCOA1
MOL011075	Shikodonin	Mineralocorticoid receptor	NR3C2
MOL001771	Poriferast-5-en-3beta-ol	Progesterone receptor	PGR
MOL001771	Poriferast-5-en-3beta-ol	Nuclear receptor coactivator 2	NCOA2
MOL002773	β-carotene	Vascular endothelial growth factor A	VEGFA
MOL002773	β-carotene	Apoptosis regulator Bcl-2	BCL2
MOL002773	β-carotene	Caspase-9	CASP9
MOL002773	β-carotene	Caspase-3	CASP3
MOL002773	β-carotene	Caspase-8	CASP8
MOL002773	β-carotene	Cytochrome P450 3A4	CYP3A4
MOL002773	β-carotene	Serum albumin	ALB
MOL002773	β-carotene	Caveolin-1	CAV1
MOL002773	β-carotene	Catenin beta-1	CTNNB1

MOL002773	β-carotene	Myc proto-oncogene protein	MYC
MOL002773	β-carotene	Caspase-7	CASP7
MOL000449	Stigmasterol	Progesterone receptor	PGR
MOL000449	Stigmasterol	Mineralocorticoid receptor	NR3C2
MOL000449	Stigmasterol	Nuclear receptor coactivator 2	NCOA2
MOL000449	Stigmasterol	Nuclear receptor coactivator 1	NCOA1
MOL000449	Stigmasterol	Prostaglandin G/H synthase 1	PTGS1
MOL000449	Stigmasterol	Aldose reductase	AKR1B1
MOL000449	Stigmasterol	Urokinase-type plasminogen activator	PLAU
MOL000449	Stigmasterol	Chymotrypsinogen B	CTRB1
MOL000449	Stigmasterol	Muscarinic acetylcholine receptor M3	CHRM3
MOL000449	Stigmasterol	Muscarinic acetylcholine receptor M1	CHRM1
MOL000449	Stigmasterol	Alpha-1A adrenergic receptor	ADRA1A
MOL000449	Stigmasterol	Muscarinic acetylcholine receptor M2	CHRM2
MOL000449	Stigmasterol	Gamma-aminobutyric acid receptor subunit alpha-1	GABRA1
MOL000354	Isorhamnetin	Prostaglandin G/H synthase 1	PTGS1
MOL000354	Isorhamnetin	Estrogen receptor	ESR1
MOL000354	Isorhamnetin	Androgen receptor	AR
MOL000354	Isorhamnetin	Peroxisome proliferator activated receptor gamma	PPARG
MOL000354	Isorhamnetin	Estrogen receptor beta	ESR2
MOL000354	Isorhamnetin	Glycogen synthase kinase-3 beta	GSK3B
MOL000354	Isorhamnetin	Trypsin-1	PRSS1
MOL000354	Isorhamnetin	Nuclear receptor coactivator 2	NCOA2
MOL000354	Isorhamnetin	Serine/threonine-protein kinase Chk1	CHEK1

MOL000354	Isorhamnetin	Aldose reductase	AKR1B1
MOL000354	Isorhamnetin	Nuclear receptor coactivator 1	NCOA1
MOL000354	Isorhamnetin	Coagulation factor VII	F7
MOL000354	Isorhamnetin	Acetylcholinesterase	ACHE
MOL000354	Isorhamnetin	Gamma-aminobutyric acid receptor subunit alpha-1	GABRA1
MOL000354	Isorhamnetin	Glutamate receptor 2	GRIA2
MOL000354	Isorhamnetin	Transcription factor p65	RELA
MOL000354	Isorhamnetin	Oxidized low-density lipoprotein receptor 1	OLR1
MOL000358	β-sitosterol	Progesterone receptor	PGR
MOL000358	β-sitosterol	Nuclear receptor coactivator 2	NCOA2
MOL000358	β-sitosterol	Prostaglandin G/H synthase 1	PTGS1
MOL000358	β-sitosterol	Muscarinic acetylcholine receptor M3	CHRM3
MOL000358	β-sitosterol	Muscarinic acetylcholine receptor M1	CHRM1
MOL000358	β-sitosterol	Muscarinic acetylcholine receptor M4	CHRM4
MOL000358	β-sitosterol	Alpha-1A adrenergic receptor	ADRA1A
MOL000358	β-sitosterol	Muscarinic acetylcholine receptor M2	CHRM2
MOL000358	β-sitosterol	Neuronal acetylcholine receptor subunit alpha-2	CHRNA2
MOL000358	β-sitosterol	Gamma-aminobutyric acid receptor subunit alpha-1	GABRA1
MOL000358	β-sitosterol	Apoptosis regulator Bcl-2	BCL2
MOL000358	β-sitosterol	Caspase-9	CASP9
MOL000358	β-sitosterol	Caspase-3	CASP3
MOL000358	β-sitosterol	Caspase-8	CASP8
MOL000358	β-sitosterol	Protein kinase C alpha type	PRKCA
MOL000358	β-sitosterol	Serum paraoxonase/arylesterase 1	PON1

MOL000392	Formononetin	Prostaglandin G/H synthase 1	PTGS1
MOL000392	Formononetin	Muscarinic acetylcholine receptor M1	CHRM1
MOL000392	Formononetin	Estrogen receptor	ESR1
MOL000392	Formononetin	Androgen receptor	AR
MOL000392	Formononetin	Peroxisome proliferator activated receptor gamma	PPARG
MOL000392	Formononetin	Alpha-1A adrenergic receptor	ADRA1A
MOL000392	Formononetin	Estrogen receptor beta	ESR2
MOL000392	Formononetin	Glycogen synthase kinase-3 beta	GSK3B
MOL000392	Formononetin	Serine/threonine-protein kinase Chk1	CHEK1
MOL000392	Formononetin	Trypsin-1	PRSS1
MOL000392	Formononetin	Acetylcholinesterase	ACHE
MOL000392	Formononetin	Peroxisome proliferator-activated receptor gamma	PPARG
MOL000422	Kaempferol	Prostaglandin G/H synthase 1	PTGS1
MOL000422	Kaempferol	Androgen receptor	AR
MOL000422	Kaempferol	Peroxisome proliferator activated receptor gamma	PPARG
MOL000422	Kaempferol	Nuclear receptor coactivator 2	NCOA2
MOL000422	Kaempferol	Trypsin-1	PRSS1
MOL000422	Kaempferol	Progesterone receptor	PGR
MOL000422	Kaempferol	Muscarinic acetylcholine receptor M1	CHRM1
MOL000422	Kaempferol	Acetylcholinesterase	ACHE
MOL000422	Kaempferol	Muscarinic acetylcholine receptor M2	CHRM2
MOL000422	Kaempferol	Gamma-aminobutyric acid receptor subunit alpha-1	GABRA1
MOL000422	Kaempferol	Coagulation factor VII	F7
MOL000422	Kaempferol	Transcription factor p65	RELA

MOL000422	Kaempferol	Inhibitor of nuclear factor kappa-B kinase subunit beta	IKBKB
MOL000422	Kaempferol	Apoptosis regulator Bcl-2	BCL2
MOL000422	Kaempferol	Activator of 90 kDa heat shock protein ATPase homolog 1	AHSA1
MOL000422	Kaempferol	Caspase-3	CASP3
MOL000422	Kaempferol	Mitogen-activated protein kinase 8	MAPK8
MOL000422	Kaempferol	Peroxisome proliferator-activated receptor gamma	PPARG
MOL000422	Kaempferol	Cytochrome P450 3A4	CYP3A4
MOL000422	Kaempferol	Cytochrome P450 1A1	CYP1A1
MOL000422	Kaempferol	Intercellular adhesion molecule 1	ICAM1
MOL000422	Kaempferol	E-selectin	SELE
MOL000422	Kaempferol	Vascular cell adhesion protein 1	VCAM1
MOL000422	Kaempferol	Cytochrome P450 1B1	CYP1B1
MOL000422	Kaempferol	Arachidonate 5-lipoxygenase	ALOX5
MOL000422	Kaempferol	Glutathione S-transferase P	GSTP1
MOL000422	Kaempferol	Aryl hydrocarbon receptor	AHR
MOL000422	Kaempferol	26S proteasome non-ATPase regulatory subunit 3	PSMD3
MOL000422	Kaempferol	Solute carrier family 2, facilitated glucose transporter member 4	SLC2A4
MOL000422	Kaempferol	Nuclear receptor subfamily 1 group I member 3	NR1I3
MOL000422	Kaempferol	Type I iodothyronine deiodinase	DIO1
MOL000422	Kaempferol	Glutathione S-transferase Mu 1	GSTM1
MOL000422	Kaempferol	Glutathione S-transferase Mu 2	GSTM2
MOL000422	Kaempferol	Aldo-keto reductase family 1 member C3	AKR1C3
MOL004350	Ruvoside_qt	Mineralocorticoid receptor	NR3C2
MOL004350	Ruvoside_qt	Nuclear receptor coactivator 1	NCOA1

MOL000492	(+)-catechin	Prostaglandin G/H synthase 1	PTGS1
MOL000492	(+)-catechin	Estrogen receptor	ESR1
MOL000492	(+)-catechin	Nuclear receptor coactivator 2	NCOA2
MOL005236	Gibberellin	GA-binding protein subunit beta-1	GABPB1
MOL006821	(-)-epigallocatechin-3-gallate	Transcription factor p65	RELA
MOL006821	(-)-epigallocatechin-3-gallate	Inhibitor of nuclear factor kappa-B kinase subunit beta	IKBKB
MOL006821	(-)-epigallocatechin-3-gallate	Interferon regulatory factor 3	IRF3
MOL006821	(-)-epigallocatechin-3-gallate	Epidermal growth factor receptor	EGFR
MOL006821	(-)-epigallocatechin-3-gallate	Vascular endothelial growth factor A	VEGFA
MOL006821	(-)-epigallocatechin-3-gallate	G1/S-specific cyclin-D1	CCND1
MOL006821	(-)-epigallocatechin-3-gallate	Apoptosis regulator Bcl-2	BCL2
MOL006821	(-)-epigallocatechin-3-gallate	Proto-oncogene c-Fos	FOS
MOL006821	(-)-epigallocatechin-3-gallate	Eukaryotic translation initiation factor 6	EIF6
MOL006821	(-)-epigallocatechin-3-gallate	Caspase-9	CASP9
MOL006821	(-)-epigallocatechin-3-gallate	Vascular endothelial growth factor receptor 1	FLT1
MOL006821	(-)-epigallocatechin-3-gallate	Urokinase-type plasminogen activator	PLAU
MOL006821	(-)-epigallocatechin-3-gallate	Retinoblastoma-associated protein	RB1
MOL006821	(-)-epigallocatechin-3-gallate	Interleukin-6	IL6
MOL006821	(-)-epigallocatechin-3-gallate	Activator of 90 kDa heat shock protein ATPase homolog 1	AHSA1
MOL006821	(-)-epigallocatechin-3-gallate	Caspase-3	CASP3
MOL006821	(-)-epigallocatechin-3-gallate	Cellular tumor antigen p53	TP63
MOL006821	(-)-epigallocatechin-3-gallate	ETS domain-containing protein Elk-1	ELK1
MOL006821	(-)-epigallocatechin-3-gallate	Involucrin	IVL
MOL006821	(-)-epigallocatechin-3-gallate	Mitogen-activated protein kinase 8	MAPK8

MOL006821	(-)-epigallocatechin-3-gallate	NF-kappa-B inhibitor alpha	NFKBIA
MOL006821	(-)-epigallocatechin-3-gallate	Ribosomal protein S6 kinase beta-1	RPS6KB1
MOL006821	(-)-epigallocatechin-3-gallate	NADPH--cytochrome P450 reductase	POR
MOL006821	(-)-epigallocatechin-3-gallate	Caspase-8	CASP8
MOL006821	(-)-epigallocatechin-3-gallate	Fatty acid synthase	FASN
MOL006821	(-)-epigallocatechin-3-gallate	Low-density lipoprotein receptor	LDLR
MOL006821	(-)-epigallocatechin-3-gallate	E3 ubiquitin-protein ligase Mdm2	MDM2
MOL006821	(-)-epigallocatechin-3-gallate	Ras-specific guanine nucleotide-releasing factor 2	RASGRF2
MOL006821	(-)-epigallocatechin-3-gallate	RAF proto-oncogene serine/threonine-protein kinase	RAF1
MOL006821	(-)-epigallocatechin-3-gallate	Bcl-2 homologous antagonist/killer	BAK1
MOL006821	(-)-epigallocatechin-3-gallate	Bcl-2-like protein 10	BCL2L10
MOL006821	(-)-epigallocatechin-3-gallate	Catechol O-methyltransferase	COMT
MOL006821	(-)-epigallocatechin-3-gallate	Serum amyloid A protein	SAA2
MOL006821	(-)-epigallocatechin-3-gallate	Protein kinase C alpha type	PRKCA
MOL006821	(-)-epigallocatechin-3-gallate	Protein kinase C epsilon type	PRKCE
MOL006821	(-)-epigallocatechin-3-gallate	Amyloid beta A4 protein	APP
MOL006821	(-)-epigallocatechin-3-gallate	Telomerase protein component 1	TEP1
MOL006821	(-)-epigallocatechin-3-gallate	Metalloproteinase inhibitor 1	TIMP1
MOL006821	(-)-epigallocatechin-3-gallate	Metalloproteinase inhibitor 2	TIMP2
MOL006821	(-)-epigallocatechin-3-gallate	Tyrosyl-DNA phosphodiesterase 1	TDP1
MOL006821	(-)-epigallocatechin-3-gallate	Glycerophosphodiester phosphodiesterase domain-containing protein 5	GDPD5
MOL006821	(-)-epigallocatechin-3-gallate	Cyclin-dependent kinase inhibitor 1C	CDKN1C
MOL006821	(-)-epigallocatechin-3-gallate	Hypoxia-inducible factor 1-alpha	HIF1A
MOL006821	(-)-epigallocatechin-3-gallate	Fos-related antigen 1	FOSL1

MOL006821	(-)-epigallocatechin-3-gal-late	Protein fosB	FOSB
MOL006821	(-)-epigallocatechin-3-gal-late	Transcription factor jun-B	JUNB
MOL006821	(-)-epigallocatechin-3-gal-late	Transcription factor jun-D	JUND
MOL006821	(-)-epigallocatechin-3-gal-late	Protein CBFA2T1	RUNX1T1
MOL006821	(-)-epigallocatechin-3-gal-late	Proliferating cell nuclear antigen	PCNA
MOL006821	(-)-epigallocatechin-3-gal-late	Vimentin	VIM
MOL006821	(-)-epigallocatechin-3-gal-late	Receptor tyrosine-protein kinase erbB-2	ERBB2
MOL006821	(-)-epigallocatechin-3-gal-late	Ribosomal protein S6 kinase alpha-1	RPS6KA1
MOL006821	(-)-epigallocatechin-3-gal-late	T-cell surface glycoprotein CD4	CD4
MOL006821	(-)-epigallocatechin-3-gal-late	Tumor necrosis factor receptor superfamily member 6	FAS
MOL006821	(-)-epigallocatechin-3-gal-late	MAP kinase-activating death domain protein	MADD
MOL006821	(-)-epigallocatechin-3-gal-late	Cytochrome P450 3A7	CYP3A7
MOL006821	(-)-epigallocatechin-3-gal-late	3-oxoacyl-[acyl-carrier-protein] synthase, mito-chondrial	OXSM
MOL006821	(-)-epigallocatechin-3-gal-late	Estrogen receptor	ESR1
MOL006821	(-)-epigallocatechin-3-gal-late	High affinity immunoglobulin epsilon receptor subunit alpha	FCER1A
MOL006821	(-)-epigallocatechin-3-gal-late	Peroxisome proliferator-activated receptor gamma	PPARG
MOL006821	(-)-epigallocatechin-3-gal-late	Toll-like receptor 4	TLR4
MOL006821	(-)-epigallocatechin-3-gal-late	Acetyl-CoA carboxylase 1	ACACA
MOL006821	(-)-epigallocatechin-3-gal-late	Transcription factor Sp1	SP1
MOL006821	(-)-epigallocatechin-3-gal-late	Sterol regulatory element-binding protein 2	SREBF2
MOL006821	(-)-epigallocatechin-3-gal-late	Caveolin-1	CAV1
MOL006821	(-)-epigallocatechin-3-gal-late	DNA (cytosine-5)-methyltransferase 3A	DNMT3A
MOL006821	(-)-epigallocatechin-3-gal-late	Interleukin-6 receptor subunit beta	IL6ST
MOL006821	(-)-epigallocatechin-3-gal-late	Cadherin-1	CDH1

MOL006821	(-)-epigallocatechin-3-gallate	Mitogen-activated protein kinase 7	MAPK7
MOL006821	(-)-epigallocatechin-3-gallate	Calcium/calmodulin-dependent protein kinase kinase 2	CAMKK2
MOL000098	Quercetin	Prostaglandin G/H synthase 1	PTGS1
MOL000098	Quercetin	Androgen receptor	AR
MOL000098	Quercetin	Peroxisome proliferator activated receptor gamma	PPARG
MOL000098	Quercetin	Nuclear receptor coactivator 2	NCOA2
MOL000098	Quercetin	Aldose reductase	AKR1B1
MOL000098	Quercetin	Trypsin-1	PRSS1
MOL000098	Quercetin	Coagulation factor VII	F7
MOL000098	Quercetin	Acetylcholinesterase	ACHE
MOL000098	Quercetin	Gamma-aminobutyric acid receptor subunit alpha-1	GABRA1
MOL000098	Quercetin	Transcription factor p65	RELA
MOL000098	Quercetin	Epidermal growth factor receptor	EGFR
MOL000098	Quercetin	Vascular endothelial growth factor A	VEGFA
MOL000098	Quercetin	G1/S-specific cyclin-D1	CCND1
MOL000098	Quercetin	Apoptosis regulator Bcl-2	BCL2
MOL000098	Quercetin	Proto-oncogene c-Fos	FOS
MOL000098	Quercetin	Eukaryotic translation initiation factor 6	EIF6
MOL000098	Quercetin	Caspase-9	CASP9
MOL000098	Quercetin	Urokinase-type plasminogen activator	PLAU
MOL000098	Quercetin	Retinoblastoma-associated protein	RB1
MOL000098	Quercetin	Interleukin-6	IL6
MOL000098	Quercetin	Activator of 90 kDa heat shock protein ATPase homolog 1	AHSA1
MOL000098	Quercetin	Caspase-3	CASP3

MOL000098	Quercetin	Cellular tumor antigen p53	TP63
MOL000098	Quercetin	ETS domain-containing protein Elk-1	ELK1
MOL000098	Quercetin	NF-kappa-B inhibitor alpha	NFKBIA
MOL000098	Quercetin	NADPH--cytochrome P450 reductase	POR
MOL000098	Quercetin	Caspase-8	CASP8
MOL000098	Quercetin	RAF proto-oncogene serine/threonine-protein kinase	RAF1
MOL000098	Quercetin	Protein kinase C alpha type	PRKCA
MOL000098	Quercetin	Hypoxia-inducible factor 1-alpha	HIF1A
MOL000098	Quercetin	Protein CBFA2T1	RUNX1T1
MOL000098	Quercetin	Receptor tyrosine-protein kinase erbB-2	ERBB2
MOL000098	Quercetin	Peroxisome proliferator-activated receptor gamma	PPARG
MOL000098	Quercetin	Acetyl-CoA carboxylase 1	ACACA
MOL000098	Quercetin	Cytochrome P450 3A4	CYP3A4
MOL000098	Quercetin	Caveolin-1	CAV1
MOL000098	Quercetin	Myc proto-oncogene protein	MYC
MOL000098	Quercetin	Cytochrome P450 1A1	CYP1A1
MOL000098	Quercetin	Intercellular adhesion molecule 1	ICAM1
MOL000098	Quercetin	E-selectin	SELE
MOL000098	Quercetin	Vascular cell adhesion protein 1	VCAM1
MOL000098	Quercetin	Prostaglandin E2 receptor EP3 subtype	PTGER3
MOL000098	Quercetin	Baculoviral IAP repeat-containing protein 5	BIRC5
MOL000098	Quercetin	Dual oxidase 2	DUOX2
MOL000098	Quercetin	Nitric oxide synthase, endothelial	NOS3
MOL000098	Quercetin	Heat shock protein beta-1	HSPB1

MOL000098	Quercetin	Maltase-glucoamylase, intestinal	MGAM
MOL000098	Quercetin	Cytochrome P450 1B1	CYP1B1
MOL000098	Quercetin	G2/mitotic-specific cyclin-B1	CCNB1
MOL000098	Quercetin	Arachidonate 5-lipoxygenase	ALOX5
MOL000098	Quercetin	Glutathione S-transferase P	GSTP1
MOL000098	Quercetin	Nuclear factor erythroid 2-related factor 2	NFE2L2
MOL000098	Quercetin	NAD(P)H dehydrogenase [quinone] 1	NQO1
MOL000098	Quercetin	Poly [ADP-ribose] polymerase 1	PARP1
MOL000098	Quercetin	Aryl hydrocarbon receptor	AHR
MOL000098	Quercetin	26S proteasome non-ATPase regulatory subunit 3	PSMD3
MOL000098	Quercetin	Solute carrier family 2, facilitated glucose transporter member 4	SLC2A4
MOL000098	Quercetin	Collagen alpha-1(III) chain	COL3A1
MOL000098	Quercetin	DDB1- and CUL4-associated factor 5	DCAF5
MOL000098	Quercetin	Nuclear receptor subfamily 1 group I member 3	NR1I3
MOL000098	Quercetin	Serine/threonine-protein kinase Chk2	CHEK2
MOL000098	Quercetin	Heat shock factor protein 1	HSF1
MOL000098	Quercetin	C-reactive protein	CRP
MOL000098	Quercetin	Runt-related transcription factor 2	RUNX2
MOL000098	Quercetin	Ras association domain-containing protein 1	RASSF1
MOL000098	Quercetin	Cathepsin D	CTSD
MOL000098	Quercetin	Insulin-like growth factor-binding protein 3	IGFBP3
MOL000098	Quercetin	Insulin-like growth factor II	IGF2
MOL000098	Quercetin	Interferon regulatory factor 1	IRF1
MOL000098	Quercetin	Receptor tyrosine-protein kinase erbB-3	ERBB3

MOL000098	Quercetin	Serum paraoxonase/arylesterase 1	PON1
MOL000098	Quercetin	Type I iodothyronine deiodinase	DIO1
MOL000098	Quercetin	Puromycin-sensitive aminopeptidase	NPEPPS
MOL000098	Quercetin	Hexokinase-2	HK2
MOL000098	Quercetin	Ras GTPase-activating protein 1	RASA1
MOL000098	Quercetin	Glutathione S-transferase Mu 1	GSTM1
MOL000098	Quercetin	Glutathione S-transferase Mu 2	GSTM2

Table S3. The negative CDOCKER interaction energy of the bioactive compounds to the core gene targets.

PDB [1] ID	Core Gene	Quercetin (kcal/ mol)	EGCG [2] (kcal/ mol)	Kaempferol (kcal/ mol)
1FOS	FOS	46.1576	57.1041	46.7322
1A52	ESR1	44.0089	49.7524	41.317
3ELJ	MAPK8	66.6726	60.6026	63.794
1VA1	SP1	20.9838	—	21.7741

[1] PDB refers to protein data bank; [2] EGCG refers to (-)-epigallocatechin-3-gallate.

Supplementary Figures

Figure S1. Effects of WGP on organ indexes.

Figure S2. Effects of WGP on serum biochemical lipid parameter LDH.

Figure S3. PPI network of WGP for anti-aging, anti-atherosclerosis and anti-fatigue using STRING database.

Figure S4. GO enrichment analysis and KEGG pathway analysis for WGP-Fatigue.

Figure S5. Molecular docking to model the interaction between ESR1 and three active ingredients of WGP (quercetin, EGCG or kaempferol).

Figure S6. Molecular docking to model the interaction between MAPK8 and three active ingredients of WGP (quercetin, EGCG or kaempferol).

Figure S7. Molecular docking to model the interaction between SP1 and two active ingredients of WGP (quercetin or kaempferol).

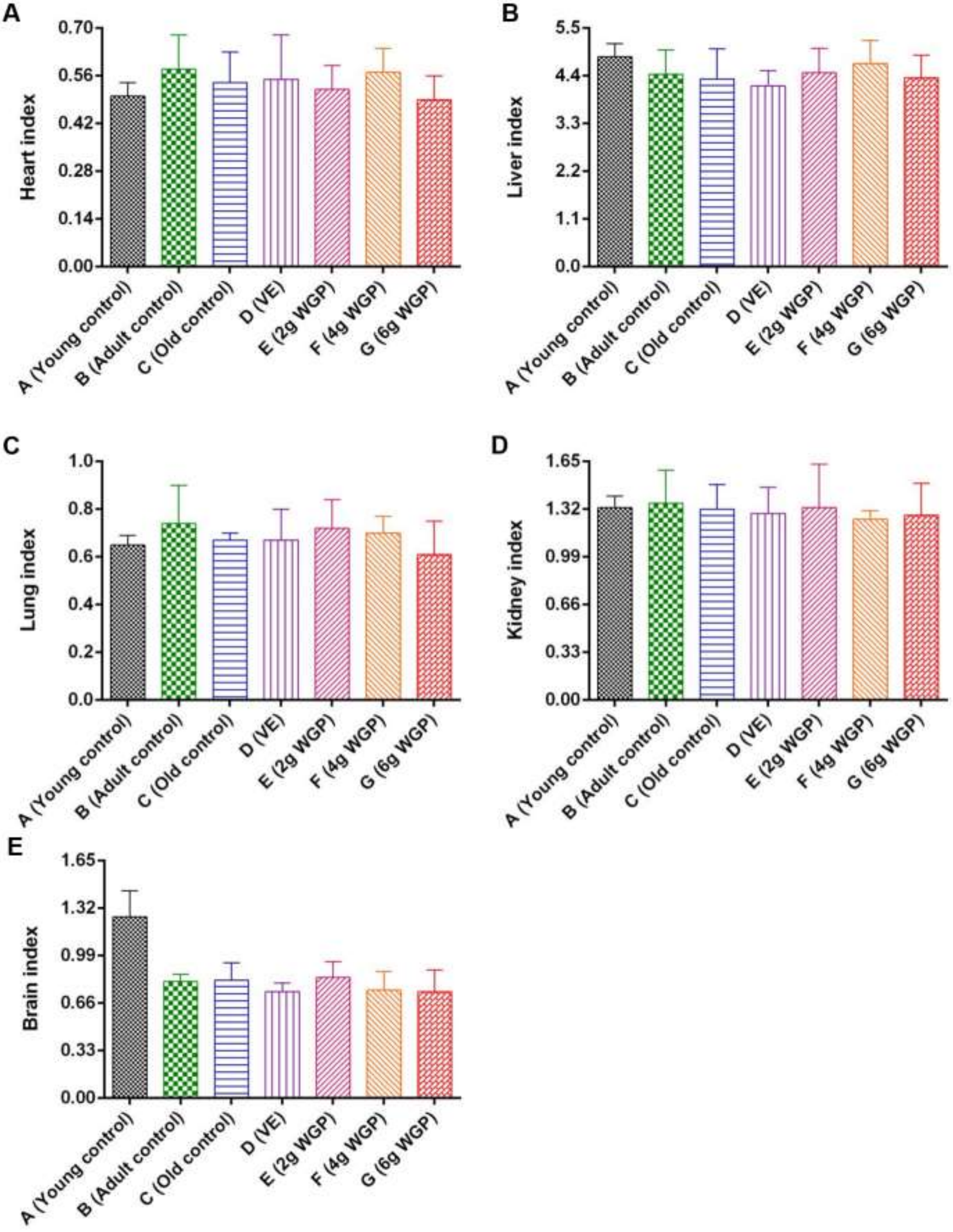

Figure S1. Effects of WGP on organ indexes. The heart index (**A**), liver index (**B**), lung index (**C**), kidney index (**D**), and brain index (**E**) of the mice treated with different WGP.

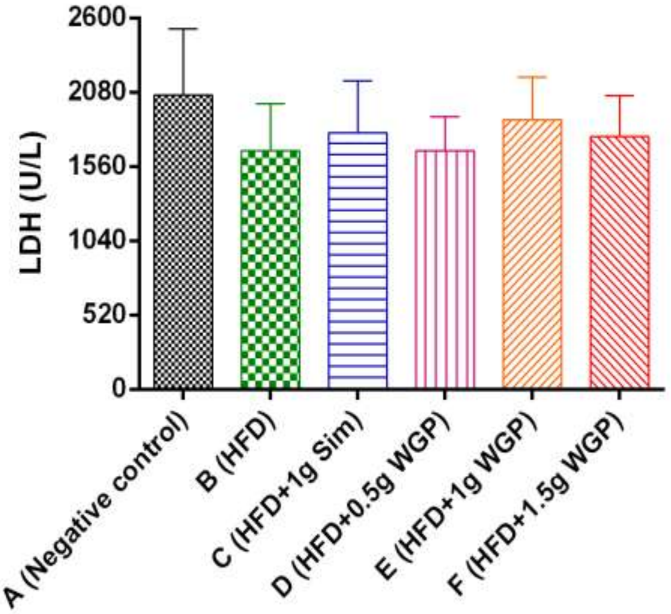

Figure S2. Effects of WGP on serum biochemical lipid parameter LDH. Data are expressed mean ± standard deviation (SD) (n = 10). Group A: negative control, old; Group B: model control, HFD-fed; Group C: treatment, 1 g simvastatin (Sim)-treated; Group D: treatment, 0.5 g WGP-treated; Group E: treatment, 1 g WGP-treated; Group F: treatment, 1.5 g WGP-treated. There are no statistically significant difference between two groups.

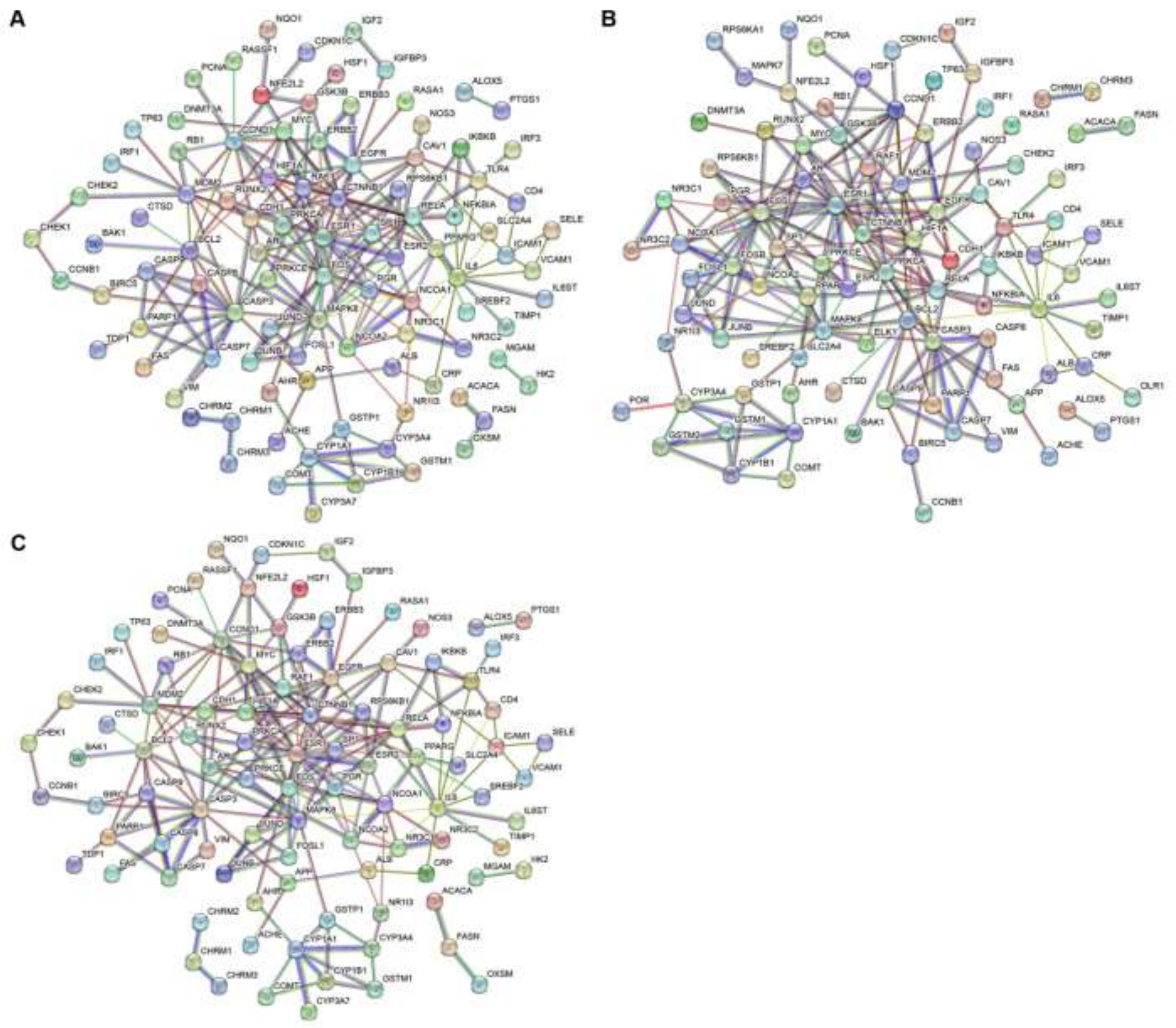

Figure S3. PPI network of WGP for anti-aging, anti-atherosclerosis and anti-fatigue using STRING database. PPI network of WGP-Aging (**A**), WGP-Atherosclerosis (**B**), and WGP-Fatigue (**C**).

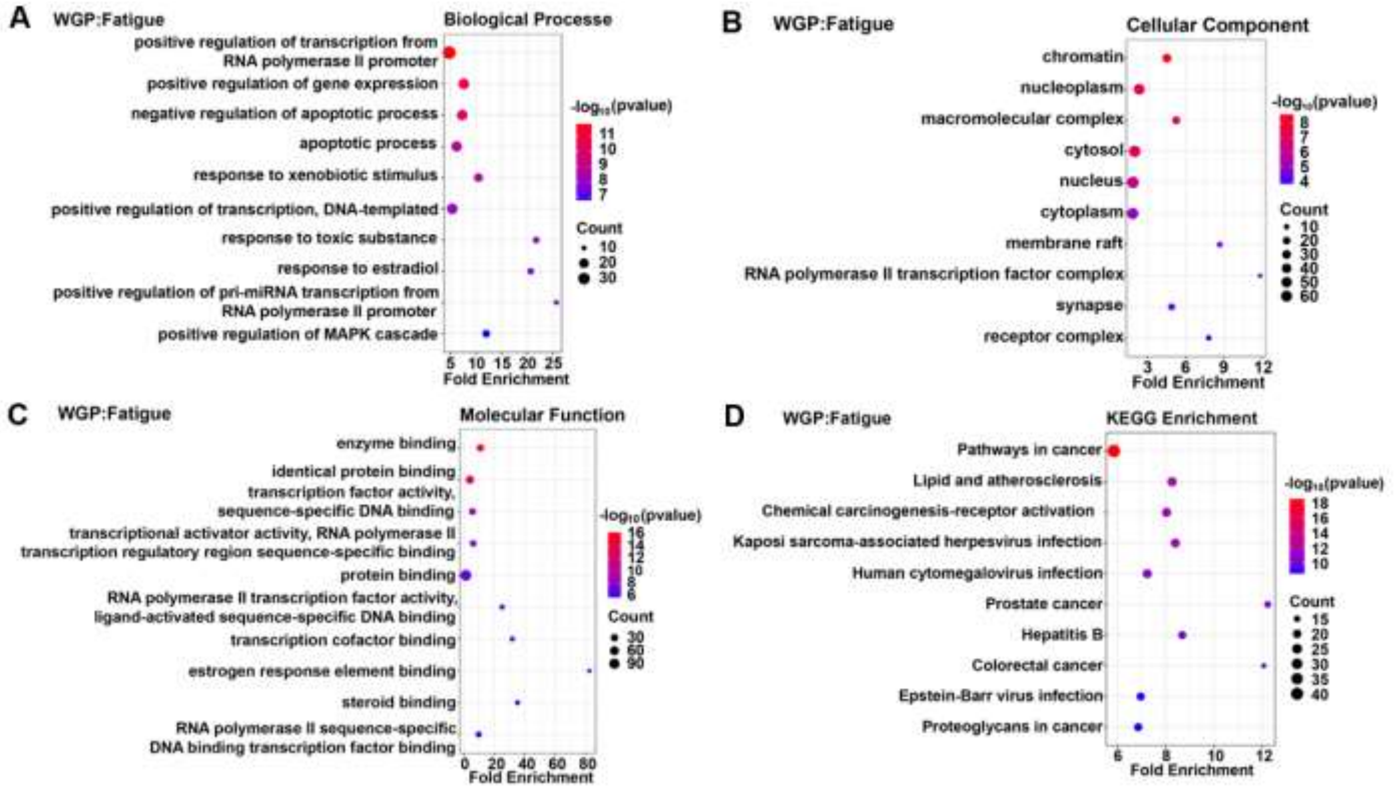

Figure S4. GO enrichment analysis and KEGG pathway analysis for WGP-Fatigue (FDR ≤ 0.05). The biological processes (top 10, (**A**)), the cellular components (top 10, (**B**)), the molecular functions (top 10, (**C**)), and the KEGG pathways (top 10, (**D**)) analysis for WGP- Fatigue. The bubble size represents the number of targets in the pathway. The bubble color indicates the magnitude of the -$\log_{10}(p)$ values.

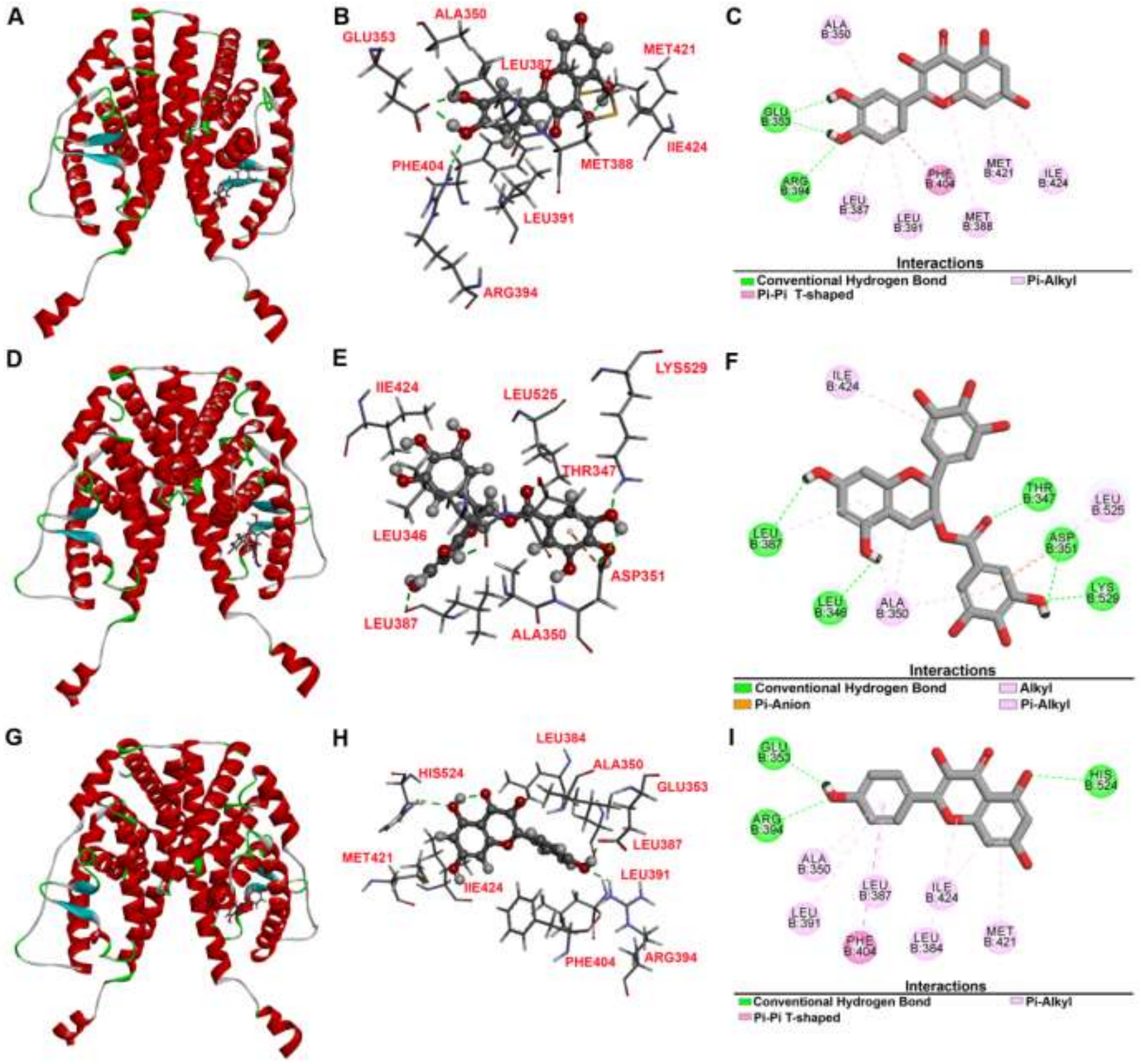

Figure S5. Molecular docking to model the interaction between ESR1 and three active ingredients of WGP (quercetin, EGCG or kaempferol). (**A**-**C**) Interaction of ESR1 and quercetin, including binding conformation of ESR1-quercetin complex (**A**), and electrical interactions of residue on ESR1 with quercetin (**B**,**C**). (**D**-**F**) Interaction of ESR1 and EGCG, including binding conformation of ESR1-EGCG complex (**D**), and electrical interactions of residue on ESR1 with EGCG (**E**,**F**). (**G**-**I**) Interaction of ESR1 and kaempferol, including binding conformation of ESR1-kaempferol complex (**G**), and electrical interactions of residue on ESR1 with kaempferol (**H**,**I**).

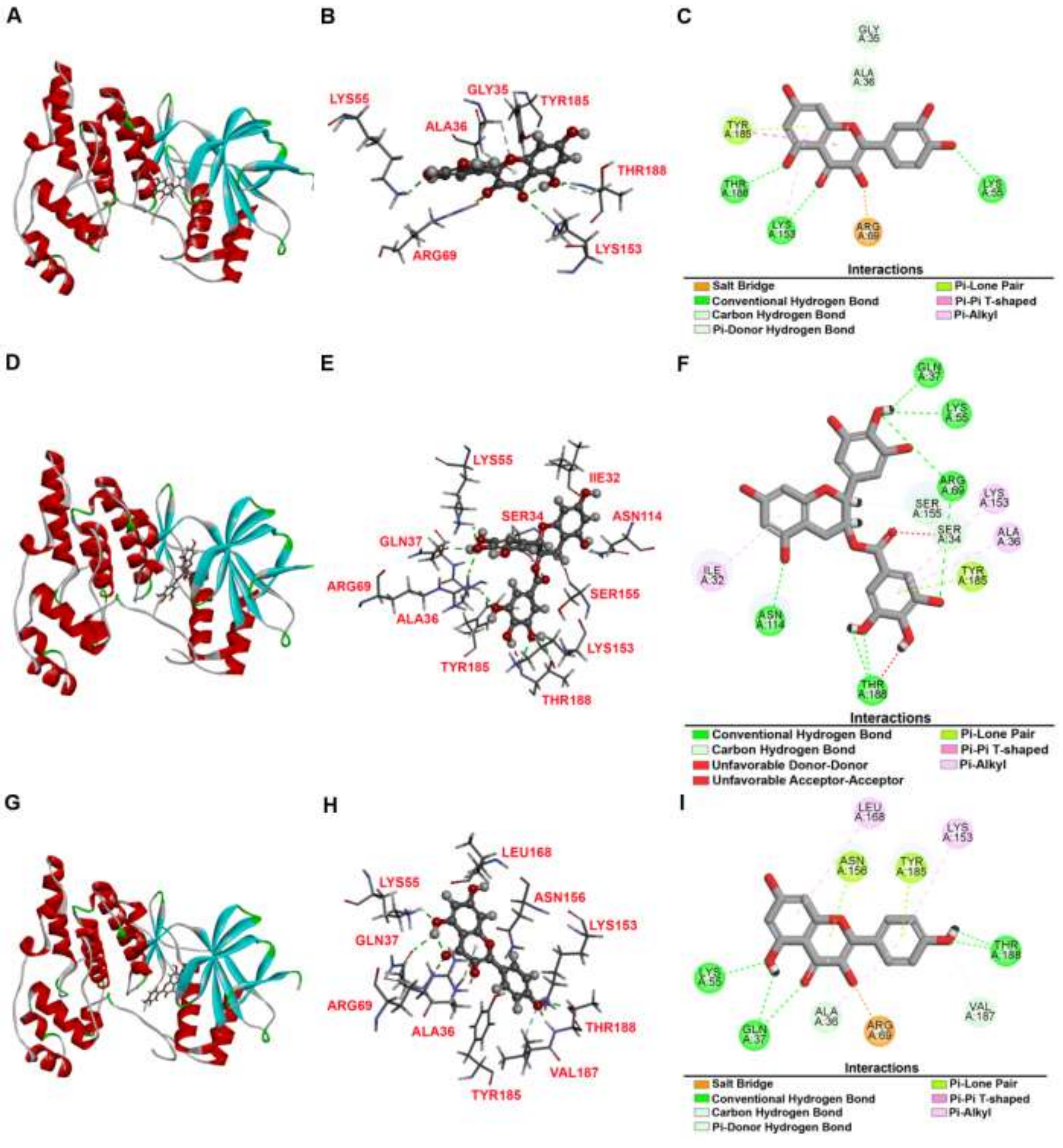

Figure S6. Molecular docking to model the interaction between MAPK8 and three active ingredients of WGP (quercetin, EGCG or kaempferol). (**A**-**C**) Interaction of MAPK8 and quercetin, including binding conformation of MAPK8-quercetin complex (A), and electrical interactions of residue on MAPK8 with quercetin (**B**,**C**). (**D**-**F**) Interaction of MAPK8 and EGCG, including binding conformation of MAPK8-EGCG complex (**D**), and electrical interactions of residue on MAPK8 with EGCG (**E**,**F**). (**G**-**I**) Interaction of MAPK8 and kaempferol, including binding conformation of MAPK8-kaempferol complex (**G**), and electrical interactions of residue on MAPK8 with kaempferol (**H**,**I**).

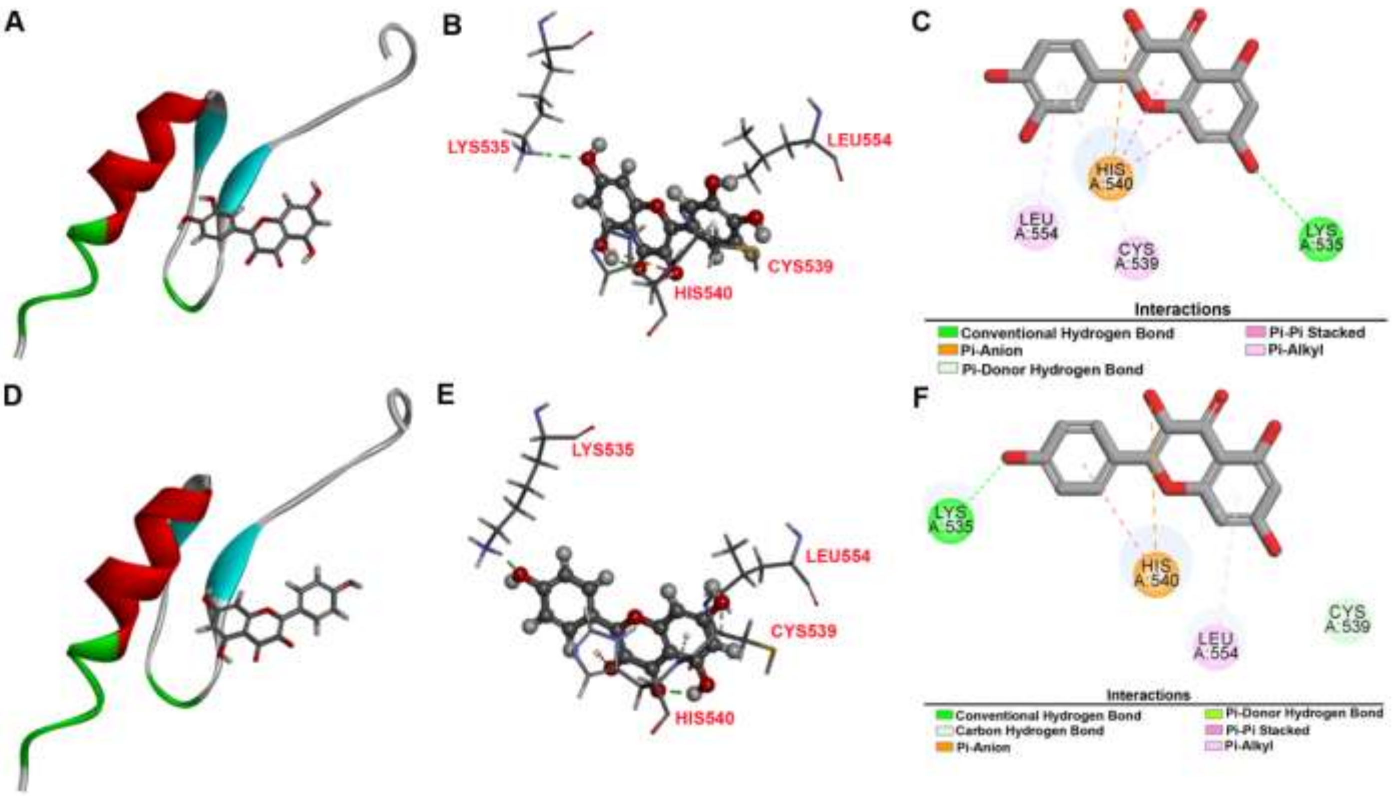

Figure S7. Molecular docking to model the interaction between SP1 and two active ingredients of WGP (quercetin or kaempferol). (**A**-**C**) Interaction of SP1 and quercetin, including binding conformation of SP1-quercetin complex (**A**), and electrical interactions of residue on SP1 with quercetin (**B**,**C**). (**D**-**F**) Interaction of SP1 and kaempferol, including binding conformation of SP1-kaempferol complex (**D**), and electrical interactions of residue on SP1 with kaempferol (**E**,**F**).
